# Systematic review and network meta-analysis of tedizolid for the treatment of acute bacterial skin and skin structure infections caused by MRSA

**DOI:** 10.1186/s12879-016-2100-3

**Published:** 2017-01-07

**Authors:** Rachael McCool, Ian M. Gould, Jacqui Eales, Teresa Barata, Mick Arber, Kelly Fleetwood, Julie Glanville, Teresa L. Kauf

**Affiliations:** 1 York Health Economics Consortium, Enterprise House, Innovation Way, University of York, Heslington, York, YO10 5NQ UK; 2 Aberdeen Royal Infirmary, Foresterhill Road, Aberdeen, AB25 2ZN UK; 3 Quantics, 28 Drumsheugh Gardens, Edinburgh, EH3 7RN UK; 4 Merck & Co., Inc., 2000 Galloping Hill Road, Kenilworth, NJ 07033 USA

**Keywords:** Acute bacterial skin and skin structure infections, Antibacterial, Complicated skin and skin structure infections, Methicillin-resistant *Staphylococcus aureus*, Network meta-analysis, Systematic review

## Abstract

**Background:**

Tedizolid, the active moiety of tedizolid phosphate, is approved in the United States, the European Union, Canada and a number of other countries for the treatment of acute bacterial skin and skin structure infections (ABSSSI) caused by certain susceptible bacteria, including methicillin-resistant *Staphylococcus aureus* (MRSA). This network meta-analysis (NMA) evaluates the comparative effectiveness of tedizolid and other antibacterials indicated for the treatment of ABSSSI caused by MRSA.

**Methods:**

Systematic review of 10 databases was undertaken to inform an NMA to estimate the relative effectiveness of tedizolid and established monotherapy comparators (ceftaroline, daptomycin, linezolid, teicoplanin, tigecycline, vancomycin) for treating MRSA-associated ABSSSI. Randomized controlled trials enrolling adults with ABSSSI or complicated skin and skin structure infections caused by suspected/documented MRSA were eligible for inclusion. Networks were developed based on similarity of study design, patient characteristics, outcome measures and available data. Outcomes of interest included clinical response at end of therapy (EOT), post-therapy evaluation (PTE) or test-of-cure assessment and treatment discontinuations resulting from adverse events (AEs). Bayesian NMA was conducted for each outcome using fixed-effects and random effects models.

**Results:**

Literature searches identified 3,618 records; 15 trials met the inclusion criteria and were considered suitable for NMA comparison. In fixed-effects models, tedizolid had higher odds of clinical response at EOT (odds ratio [OR], 1.7; credible interval, 1.0, 3.0) and PTE than vancomycin (OR, 1.6; credible interval, 1.1, 2.5). No differences in odds of clinical response at EOT or PTE were observed between tedizolid and other comparators. There was no evidence of a difference among treatments for discontinuation due to AEs. Results from random effects and fixed-effects models were generally consistent.

**Conclusions:**

Tedizolid was superior to vancomycin for clinical response at EOT and PTE. There was no evidence of a difference between tedizolid and other comparators and no evidence of a difference between tedizolid and all comparators when evaluating discontinuation due to AEs. These findings suggest that tedizolid provides an alternative option for the management of serious skin infections caused by suspected or documented MRSA. This study is subject to the limitations inherent in all NMAs, and the results should be interpreted accordingly.

**Electronic supplementary material:**

The online version of this article (doi:10.1186/s12879-016-2100-3) contains supplementary material, which is available to authorized users.

## Background

Acute bacterial skin and skin structure infection (ABSSSI) is a relatively new classification of skin infections introduced by the US Food and Drug Administration (FDA) in 2010 [1]. ABSSSI is defined as *‘a bacterial infection of the skin with a lesion size area of at least 75 cm* ^*2*^ *(lesion size measured by the area of redness, edema, or induration)*’ and includes the following infection types: cellulitis/erysipelas, wound infection and major cutaneous abscess [1]. These infections are also encompassed by the definitions of ‘complicated skin and soft tissue infections’ (cSSTI) or ‘complicated skin and skin structure infections’ (cSSSI), but cSSTI/cSSSI classifications also extend to other types of skin infections, such as diabetic foot ulcers.

cSSTI and ABSSSI are among the most rapidly increasing reasons for hospitalization in the United States [2]. Common bacterial pathogens causing ABSSSI are *Staphylococcus aureus*, including methicillin-resistant *S. aureus* (MRSA), and *Streptococcus pyogenes* [1, 3]. MRSA is a significant cause of both health care–associated and community-associated skin and soft tissue infections [4]. It is associated with worse outcomes and higher costs of care than other pathogens, such as methicillin-susceptible *S. aureus* (MSSA), not only for skin and soft tissue infections but also for pneumonia, bacteraemia and diabetic foot infections [5].

Practice guidelines for the diagnosis and management of skin and soft tissue infections published in 2005 suggest that in specific cases of *S. aureus* infection, clinicians should assume that the organism is methicillin resistant because of the high prevalence of community-associated MRSA strains and, therefore, use agents effective against MRSA [6]. Updated guidelines, published in 2014, place an emphasis on the importance of establishing the cause of the infection and considering pathogen-specific and local resistance patterns due to the emergence of resistance to many commonly used treatment agents [7]. Standard therapy for MRSA infections has historically been vancomycin. However, the efficacy of vancomycin has increasingly been called into question, with concerns over its slow bactericidal activity and the emergence of less susceptible and resistant strains [5]. At the time of this study (February 2014), alternative available treatments for MRSA included teicoplanin, tigecycline, ceftaroline, daptomycin, telavancin and linezolid [3, 5, 8]; in the meantime, oritavancin and dalbavancin also entered the marketplace.

Tedizolid phosphate is a novel, potent oxazolidinone prodrug that is rapidly converted to microbiologically active tedizolid. Tedizolid binds to the bacterial 50S ribosomal subunit to inhibit protein synthesis, resulting in broad *in vitro* activity against Gram-positive pathogens, including MRSA and selected strains resistant to vancomycin or linezolid [9, 10]. Tedizolid is approved in a number of countries, including the United States, Canada and those of the European Union, for the treatment of ABSSSI caused by certain susceptible bacteria including MRSA [1]. Two phase 3 randomized controlled trials (RCTs) have been conducted that compared tedizolid with linezolid, but, at the time of our study, there were no other head-to-head comparisons of tedizolid with any other relevant comparators.

In light of the limited amount of head-to-head data and to explore the comparative effectiveness of tedizolid, we conducted a systematic review and network meta-analysis (NMA) to compare the effectiveness of tedizolid with other, established therapies for treating serious skin infections caused by MRSA.

## Methods

The systematic review was conducted according to the principles of systematic reviewing as set out in guidance from the Centre for Reviews and Dissemination [11] and the National Institute for Health and Care Excellence (NICE) [12]. Relevant trials were identified and selected according to a systematic review protocol (Additional file 1: Appendices) and process, and reporting conformed to the Preferred Reporting Items for Systematic Reviews and Meta-Analyses (PRISMA) guidelines [13]. This protocol has not been registered.

### Eligibility criteria

Eligible studies were RCTs of any size and duration, published in English, that evaluated tedizolid, vancomycin, linezolid, daptomycin, teicoplanin, tigecycline, ceftaroline or telavancin for the treatment of MRSA-associated ABSSSI in adults with suspected or documented MRSA-ABSSSI or cSSSI.

Studies that compared any of the above treatments of interest as monotherapy were eligible for inclusion in the NMA, but studies assessing combinations of treatments were not eligible. Included studies could be of any treatment duration and any length of follow-up. Outcomes of interest to this review were clinical response rate at early assessment (48–72 h) and at test of cure (TOC) or similar endpoint, rates of nephrotoxicity and serious adverse events (AEs) leading to treatment discontinuation. Full details of study eligibility and the outcomes assessed can be found in the online Additional file 1: Appendix A. Exclusion criteria are presented in Appendix C.

### Study identification and selection

The search strategy was structured to search for two concepts: (ABSSSI or cSSSI) AND (named comparators) AND RCTs. The RCT filter was based on the Cochrane Highly Sensitive Search Strategy for identifying randomized trials in MEDLINE: sensitivity-maximizing version (2008 revision), Ovid format [14].

Searches were conducted in February 2014 in a range of relevant databases of published research, as recommended by systematic review guidelines. The search focused on identifying fully published reports; searches for conference abstracts were not conducted. Ten databases were searched: MEDLINE In-Process & Other Non-Indexed Citations and MEDLINE (via OvidSP), EMBASE (via OvidSP), Science Citation Index Expanded (via Web of Science), Cochrane Database of Systematic Reviews (via Cochrane Library/Wiley), Cochrane Central Register of Controlled Trials (via Cochrane Library/Wiley), Database of Abstracts of Reviews of Effects (via Cochrane Library/Wiley), Health Technology Assessment Database (via Cochrane Library/Wiley), ClinicalTrials.gov (via http://www.clinicaltrials.gov), International Clinical Trials Registry Platform (via http://apps.who.int/trialsearch/) and metaRegister of Controlled Trials (via http://www.controlled-trials.com/mrct/). The searches were not limited by date range or language. Details of the search strategy for MEDLINE are reported in the online Additional file 1: Appendix B; this strategy was translated appropriately for other databases.

Records were assessed by one reviewer for relevance on the basis of information provided in the title and abstract, and a sample of records was checked by a second reviewer; any disagreements were discussed with a third reviewer. Full-text copies of potentially relevant documents were obtained and evaluated against the eligibility criteria defined in the protocol (Appendix A). The full-text assessment was undertaken by two independent reviewers, with a third reviewer to adjudicate any disagreements. Studies that were excluded following full-text assessment are reported in the online Additional file 1: Appendix C, along with the reason for exclusion.

### Determining the suitability of indirect comparisons

Following the identification of relevant studies, theoretical networks were produced based on the interventions and comparators in each trial. Network diagrams showing which of the treatments and comparator treatments were linked for each outcome were developed; any assumptions made to connect the networks were clearly described, and the implications of the assumptions were discussed.

To assess the similarity of studies, we adapted the guidance produced by the Australian Pharmaceutical Benefits Advisory Committee (PBAC) [15] on best practice for the conduct of indirect and mixed-treatment comparisons. The PBAC guidance suggests that the similarity of the studies in each network should be assessed by evaluating the following elements: quality of methods used in conducting randomized trials, confounding factors in relation to participant populations, confounding factors in relation to circumstances, similarity of treatments (common reference and interventions) and similarity of outcomes and measures.

### Data extraction

Data extraction of each of the included studies in the systematic review was carried out by two independent reviewers using a standardized data extraction form to capture these similarity elements in DistillerSR software. A third reviewer compared the extractions and highlighted any disagreements for discussion.

Key study characteristics, populations, treatments and efficacy and safety outcomes data were used to determine whether studies were sufficiently similar to combine in an NMA and to assess which studies provided usable data for the outcomes of interest. The following outcomes were selected as priorities for the NMA: clinical response at early assessment (48-72 h after the first dose of study medication and investigator assessed), end of therapy (EOT) evaluation, post-therapy evaluation (PTE) or TOC, AEs leading to discontinuation and nephrotoxicity.

### Network meta-analysis

Networks were developed based on similarity in study design, outcome measures and available data. NMA methods were informed by the good research practice guidelines developed by the International Society for Pharmacoeconomics and Outcomes Research task force [16] and PBAC [15].

Differences between treatments within studies were analyzed in the context of a network of treatment comparisons. For each outcome, the NMA synthesized the results across the studies to give overall estimates of the odds ratios for each pair of treatments within the network. Standard Bayesian methodology for NMA was applied to all outcomes. All analyses were conducted using WinBUGS version 1.4.3 [17] and R version 3.1.1 [18]. The package ‘R2WinBUGS’ was used to run WinBUGS from within R [19]. The WinBUGS code for the models is provided in the online Additional file 1: Appendix D.

Both fixed-effects and random effects models were fitted to the data. Due to the moderate to limited information available to estimate the between-study variance, only results for the fixed-effects models are discussed in the main body of the paper.

Statistical heterogeneity was assessed for each outcome, for each pairwise treatment comparison informed by at least two trials. Heterogeneity was measured by the I^2^ statistic, where I^2^ values of 25%, 50% and 75% were considered to indicate low, moderate and high heterogeneity, respectively [20]. Inconsistency—the lack of agreement between direct and indirect evidence in an NMA—was also assessed for each loop in the networks. Inconsistency was assessed by the Bucher method, as described in the NICE Decision Support Unit Technical Support Document [21].

## Results

### Literature review and network development

#### Included studies

**Fig. 1:**
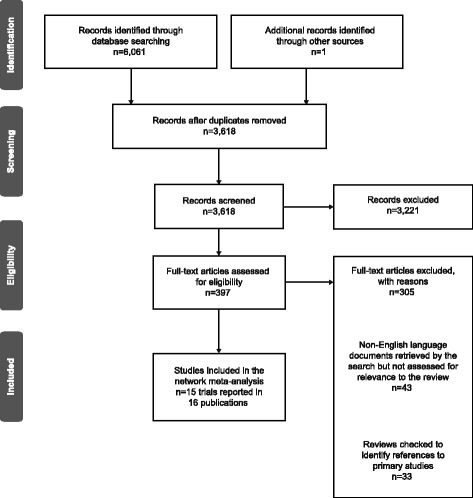
PRISMA flow diagram [13] for the review and NMA

Fifteen trials (16 reports) met the inclusion criteria for the systematic review and NMA. A flow diagram of the numbers of studies included and excluded at each stage of the selection process is provided in Fig. 1.

#### Feasibility assessment

Following identification of relevant studies, the similarity of the trials was assessed to determine whether it would be appropriate to combine the trials in an NMA. All 15 trials evaluated in the feasibility assessment were included in the NMA.

#### Determining the similarity of studies for indirect comparisons

The interventions assessed in the 15 eligible trials are summarized in Table 1. A summary of the similarity of trials for each criterion in the PBAC tool, highlighting any key differences, is presented in the following sections, and the detailed assessment is presented in the online Additional file 1: Appendix E.

**Table 1:** Summary of treatments assessed in each trial and the networks possible with the available data

Study	Treatments								Outcomes Assessed						
	Tedizolid	Tigecycline	Ceftaroline	Teicoplanin	Linezolid	Vancomycin	Daptomycin	Telavancin	EOT – all	EOT – ITT/mITT only	PTE/TOC – all	PTE/TOC – ITT/mITT only	EOT/PTE/TOC – MRSA only	Discontinuation due to AE – all	PTE/TOC – MRSA only (post hoc)
Aikawa et al., 2013 [22]						x	x				x	x	x	x	x
Corey et al., 2010 [35]			x			x^a^									x
Evers et al., 2013 [29]							x	x			x	x			
Florescu et al., 2008 [32]		x				x					x	x	x		x
Itani et al., 2010 [23]					x	x			x	x	x	x	x		x
Kohno et al., 2007 [24]					x	x			x		x		x		x
Lin et al., 2008 [34]					x	x			x		x				
Moran et al., 2014 [9]	x				x				x	x	x	x		x	
Pertel et al., 2009 [30]						x	x				x	x		x	
Prokocimer et al., 2013 [10]	x				x				x	x	x	x	x	x	x
Sharpe et al., 2005 [25]					x	x					x	x	x		x
Stevens et al., 2002 [26]					x	x					x	x	x		x
Stryjewski et al., 2008 [33]						x		x			x	x	x	x	x
Talbot et al., 2007 [31]			x			x			x		x	x	^b^	x	x
Weigelt et al., 2005 [27]					x	x					x	x		x	
Wilcox et al., 2004 [28]				x	x				x	x					

*AE* adverse event, *EOT* clinical response at the end of treatment, *ITT* intention to treat, *mITT* modified intention to treat, *MRSA* methicillin-resistant *Staphylococcus aureus*, *PTE* clinical response at the post-treatment evaluation, *TOC* clinical response at the test of cure

^a^In the Corey 2010 study, vancomycin was given with or without aztreonam

^b^ For clinical response at EOT/PTE/TOC, Talbot 2007 was the only study of ceftaroline. Only five patients on each arm had confirmed MRSA. Hence, for this network, data were insufficient to estimate comparisons with ceftaroline (the model did not converge), and Talbot 2007 was excluded. It was included, however, in the post hoc analysis because for this network there were other studies with ceftaroline

#### Trial design and quality

All included trials were reported to be RCTs. However, the type and degree of blinding or allocation concealment was variable across trials, representing a potential source of heterogeneity. Seven trials were open label [22–28], three were single blind [29–31] (two in which the assessors were blinded [30, 31]), four were double blind [9, 10, 32, 33] and one did not report details of blinding [34].

The exact outcome assessed for each trial may be a further source of heterogeneity because the precise definition of clinical response varied from study to study.

Across the trials, similar treatment time periods were assessed and the follow-up time was generally similar for the EOT and end-of-study time points. With one exception [34], the proportion of patients lost to follow-up or missing data was similar across treatment arms within a trial, and these proportions were generally similar across trials (with the exception of Wilcox et al 2004 and Stryjewski et al, 2007 [28, 33]). Large proportions and/or different proportions lost to follow-up between treatment arms can be a source of attrition bias.

#### Analysis population

There was variation in the population groups analyzed, and several trials reported data for a number of populations. A number of trials assessed the intention-to-treat (ITT) population, but most trials assessed a modified ITT (mITT) population comprising only patients who received a baseline amount of a drug and/or patients who were evaluable (definitions varied across trials) or those who followed the protocol (to varying degrees). Most studies were conducted in the United States (*n* = 11); 3 studies were conducted in Asia and 2 in Europe. The various populations defined in the trials and for whom data are reported are presented in the online Additional file 1: Appendix E, section E.4.

#### Participant populations

Across the 15 trials and between treatment arms, populations were broadly similar in terms of eligible age, actual age and sex. Diagnostic workup was generally well reported by the trials, but there were some differences in the criteria used to determine infection and/or MRSA infection likely due to the variability of presentation of infection symptoms.

Variability existed between the trials in the proportions of patients with cellulitis, wounds, abscess and other types of infection, each varying from approximately 10% to 50%. In general, variability existed between trials rather than between treatment arms within the same trial. Some studies, particularly those that included patients with non-SSSI infections, did not report details of infections, and type of infection was not taken into consideration in the NMA. Trials also varied in the proportion of patients with confirmed MRSA. This variability reflected the differing scopes of the trials, some of which focused on MRSA [32] whereas others focused on a wider scope of infective pathogens [31]. With the exception of the variability in the infection details between studies, the baseline population characteristics of the included trials did not present major heterogeneity for this proposed NMA.

#### Common treatment arms

Similarity assessments of the treatment arms common to more than one study, and that feature in the NMA, were also undertaken. For most treatments, the treatment dose, schedule and duration were similar across trials and did not present heterogeneity issues for the potential NMA. The exception was vancomycin, for which the treatment duration was 7 to 14 days in seven studies and up to 21 or 28 days in the other four studies, three of which included patients with a range of infections (i.e. not restricted to skin infections), which could explain the longer treatment durations [24, 32, 34]. There were also some differences in the administration methods for tedizolid and linezolid because both treatments could be administered orally or intravenously.

#### Conclusions of the feasibility assessment

The primary network analyzed included all trials with data for the particular outcome (network designated as ‘all trials’). However, the difference in the analysis populations across the trials was considered a notable source of heterogeneity. Therefore, these differences were explored further in a sensitivity analysis. Where data were reported for multiple populations in one trial, data for the ITT population, or those of the population that most closely resembled the ITT population, were used. A second network was then analyzed that included only the trials reporting data for an ITT or a modified population (network identified as ‘ITT/mITT’).

The primary population of interest to this review was patients with MRSA-associated ABSSSI*.* Several of the trials, however, included patients with infections caused by other Gram-positive pathogens*.* For this reason, a third network was also explored for each outcome that included only trials of patients with confirmed MRSA or trials reporting data for subgroups of patients with confirmed MRSA (network identified as ‘MRSA only’).

Overall, though there were some differences in the study methods and patient populations included in the identified trials, the studies were deemed similar enough for reasonable estimates to be derived of the comparative efficacy of the treatments using methods of indirect treatment comparison.

### Network meta-analysis

**Fig. 2:**
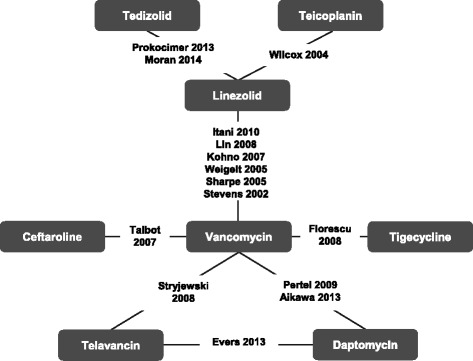
Network diagram of studies of ABSSSI treatment

Fifteen studies contributed to the NMA. The full network diagram with all the included trials is presented in Fig. 2. The outcomes of clinical response at EOT and PTE/TOC and AEs leading to discontinuation had connected networks and were selected for analysis in the NMA. It was not possible to create a connected network comparing tedizolid and the comparators of interest for clinical response at early assessment or nephrotoxicity. The networks varied depending on the data available in each trial; none of the NMA included all 15 trials. In total, 14 studies contributed to the all-trials network, 12 studies to the ITT/mITT network and eight studies to the MRSA-only network. Table 1 identifies the treatments included in each study and illustrates which studies are included in each network.

#### Assessment of heterogeneity and inconsistency

No significant heterogeneity was found between any of the pairwise comparisons for clinical response at EOT and discontinuations due to AEs. For clinical response at PTE, the linezolid versus vancomycin comparison showed moderate to high heterogeneity between the trials.

Inconsistency could be checked only for clinical response at PTE because this was the only network with a closed loop (between the vancomycin, telavancin and daptomycin treatments). No inconsistency was found.

Table 2 shows the results for tedizolid and each of the seven comparator treatments; credible intervals (CrI) that did not cross 1 and favoured tedizolid are in bold text.

**Table 2:** Fixed-effects results of NMA comparing tedizolid with each of the seven comparator drugs (odds ratios [95% credible intervals])

Outcome	Comparator Drug						
	Ceftaroline	Daptomycin	Linezolid	Teicoplanin	Telavancin	Tigecycline	Vancomycin
Clinical response at the end of treatment							
All trials	0.7 [0.0, 30.6]	NA	1.0 [0.7, 1.3]	2.2 [0.6, 9.0]	NA	NA	**1.7** **[1.0, 3.0]**
ITT/mITT only	NA	NA	1.0 [0.7, 1.3]	2.2 [0.6, 9.0]	NA	NA	1.5 [0.8, 2.6]
Clinical response at PTE/TOC							
All trials	1.0 [0.3, 3.5]	1.4 [0.5, 3.8]	1.0 [0.7, 1.4]	NA	1.4 [0.9, 2.3]	3.2 [0.8, 16.9]	**1.6** **[1.1, 2.5]**
ITT/mITT only	1.0 [0.3, 3.5]	1.4 [0.5, 3.8]	1.0 [0.7, 1.4]	NA	1.4 [0.9, 2.3]	3.2 [0.8, 16.7]	**1.6** **[1.1, 2.5]**
MRSA only	NA	2.1 [0.4, 13.4]	1.0 [0.4, 2.3]	NA	1.1 [0.4, 3.0]	3.2 [0.7, 20.0]	1.6 [0.7, 4.0]
Post hoc sensitivity analysis							
MRSA only	1.2 [0.4, 3.6]	2.1 [0.4, 13.5]	1.0 [0.4, 2.3]	NA	1.1 [0.4, 3.0]	3.2 [0.7, 20.0]	1.6 [0.7, 4.0]
Discontinuation due to AE							
All trials	0.3 [0.0, 7.1]	0.8 [0.0, 45.8]	0.5 [0.1, 1.9]	NA	0.3 [0.1, 1.3]	NA	0.4 [0.1, 1.8]

Bold text indicates CrI that do not cross 1 and favour tedizolid

*AE* adverse event, *CRI* credible intervals, *EOT* end of treatment, *ITT* intention to treat, *mITT* modified intention to treat, *MRSA* methicillin-resistant *Staphylococcus aureus*, *NA* not available, *PTE* post-treatment evaluation, *TOC* test of cure

#### Clinical response at end of treatment

**Fig. 3:**
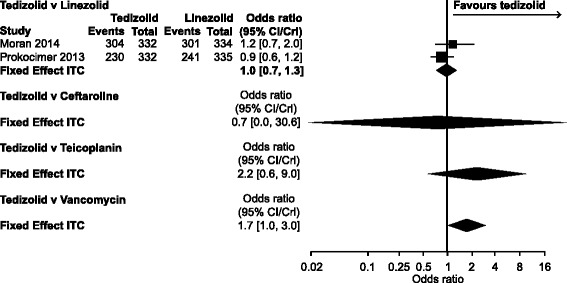
Clinical response at the end of treatment: all trials. Odds ratios (fixed-effects model)

Results for two networks, corresponding to two different populations, were estimated: all trials and ITT/mITT (Table 2, Fig. 3). The NMA results for all trials suggested that tedizolid was superior to vancomycin (odds ratio [OR], 1.7; CrI, 1.0, 3.0). There was no evidence of a difference between tedizolid and any of the other comparators assessed for the all-trials and ITT/mITT networks.

Similar results were found for the random effects models. Based on all trials, the tedizolid versus vancomycin estimate was similar, but the CrI was wider and included 1 (online Additional file 1: Appendix F). Limited information was available to estimate the between-study variance for the random effects model, which contributes to the wider CrI estimated.

#### Clinical response at the post-treatment evaluation or test of cure

**Fig. 4:**
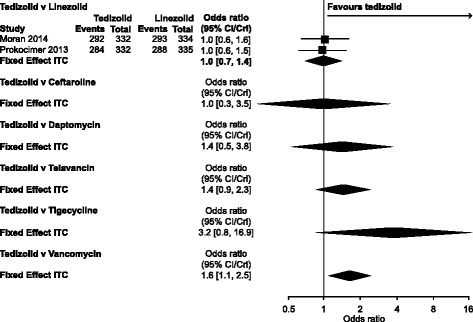
Clinical response at the post-treatment evaluation or test of cure: all trials. Odds ratios (fixed-effects model)

Results for three networks, corresponding to different populations, were estimated: all trials, ITT/mITT and MRSA only (Table 2, Fig. 4). Results of the fixed-effects model in both the all-trials and the ITT/mITT networks suggest that the odds of a clinical response at PTE or TOC were higher for tedizolid than for vancomycin (OR, 1.6; CrI, 1.1, 2.5; Fig. 4). Linezolid was also found to be superior to vancomycin and telavancin in these networks. For the MRSA-only network, linezolid was found to be superior to vancomycin. However, given that significant heterogeneity was found for the linezolid versus vancomycin comparison for all three networks, conclusions should be drawn with care. There was no evidence of a difference between any of the other comparisons assessed.

#### Post hoc sensitivity analysis

After the analysis was completed, it became clear that the exclusion of combination therapies led to exclusion of a known publication for which pathogen-specific results were available. A post hoc analysis was conducted to facilitate a comparison with ceftaroline and included a study that evaluated ceftaroline monotherapy compared with a combination treatment arm (vancomycin with and without aztreonam) [35]. This post hoc analysis was conducted for the clinical response at PTE/TOC and for the MRSA-only population. The results were similar to those obtained for the main analysis. There was no evidence of a difference between tedizolid and the other treatments.

#### Discontinuation due to adverse events

**Fig. 5:**
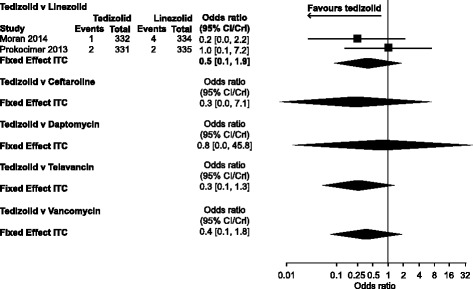
Discontinuation due to adverse events: all trials. Odds ratios (fixed-effects model)

There was no evidence of a difference between any of the treatments for discontinuation due to AEs: all comparisons had CrI that included 1 (Table 2, Fig. 5).

## Discussion

This systematic review and NMA evaluated the comparative effectiveness of tedizolid and other, established antibacterial agents indicated for treatment of ABSSSI caused by MRSA. Given the expense and difficulties of conducting further RCTs to directly compare the increasing variety of agents available for treating serious MRSA infections, it is opportune that we now have robust methods through NMA for comparing the relative efficacy of new and established agents by indirect means. A previous NMA of antibacterials used to treat MRSA infection was published before tedizolid was approved [36]; however, the challenges associated with treating ABSSSI in the era of resistant bacteria mean that up-to-date evaluations of new treatments are critical to inform treatment decisions. In the current analysis, tedizolid was superior to vancomycin for clinical response at EOT (all-trials data); there was no evidence of a difference between tedizolid and linezolid, ceftaroline or teicoplanin. For clinical response at PTE, tedizolid was superior to vancomycin, and there was no evidence of a difference compared with linezolid, daptomycin, tigecycline, ceftaroline and telavancin. The lack of a difference between the latter antibacterials is consistent with findings previously reported by Bally et al [36], which resulted from an NMA conducted in the same setting before tedizolid data were available. That analysis also suggested that some antibacterial agents, including the oxazolidinone linezolid (to which tedizolid was shown to be non-inferior in terms of efficacy), were more effective and safer than vancomycin for treating hospital patients with cSSTI caused by MRSA. Tedizolid was found to be equivalent to all comparators when evaluating discontinuation due to AEs.

This study is subject to the limitations inherent to all NMAs in terms of the quality of included studies, publication bias and limited data [37], and the results should be interpreted accordingly. Flaws in the design, conduct and analysis of RCTs can lead to bias and raise questions about the validity of the studies’ findings. In this review, a similarity assessment of studies eligible for inclusion in the networks was undertaken, as was a detailed assessment of the risk of bias for each trial identified. The included trials varied in design and quality: some were double-blind but others were not blinded, and, in general, limited information was reported on methods of randomization, sequence generation and sequence allocation. Results of the NMA should be considered in light of these uncertainties around bias. The trials also differed in terms of definitions of clinical response, and information regarding how AEs were defined was limited. It was assumed that these factors would be similar for the purpose of these analyses. Most trials did not explicitly report methods used to account for missing data, so the impact of different methods cannot be assessed.

Several other limitations of this NMA should be noted when evaluating the results. Some studies, particularly those that included non-SSSI infections, did not report details of the infection site. Furthermore, the trials differed in the relative proportions of skin infection type (i.e. cellulitis, wounds, abscesses, other infections). In addition, the analysis included trials assessing outcomes in ABSSSI and cSSSI despite their different definitions, treatment approaches and treatment outcomes.

The trials also varied in the proportion of patients with confirmed MRSA. This variability reflects the differing scopes of the trials, some of which focused exclusively on suspected or confirmed MRSA, whereas others had a wider scope of infective pathogens. Although the target population for this review was patients with MRSA-associated skin infections, the causative pathogen generally was unknown at trial entry; therefore, the inclusion criteria for this review required a ‘suspicion’ of MRSA rather than confirmed MRSA. Thus, where possible for each outcome, a network including only MRSA populations was also assessed. However, conclusions for the MRSA-only population should be drawn with care because this encompassed an analysis group of limited size. Results for this network have been estimated using only eight studies, whereas estimates for the all-trials and ITT/mITT networks for the clinical response at PTE were informed by 14 and 12 studies, respectively.

Because of the limited number of studies contributing to each network, a pragmatic approach was adopted whereby trials were included regardless of minor differences in outcome definitions and analysis methods. It was assumed that the differences in definitions and methods would not influence the relative treatment effects. These assumptions should be taken into consideration when assessing the strength of the results.

## Conclusions

Infections due to MRSA pose a considerable challenge worldwide and are associated with increased mortality, morbidity and healthcare costs. Therefore, identification of optimal treatment strategies is of the utmost importance, particularly in light of the declining efficacy of standard therapy. NMA is a useful analytical method that allowed us to study the comparative efficacy and safety of tedizolid and all established MRSA treatments even in the absence of head-to-head trials for many of these agents. This NMA showed that tedizolid may provide an alternative option for the management of serious skin infections suspected or documented to be caused by MRSA.

## Additional file

Additional file 1: Appendix A. Eligibility criteria. **Appendix B.** MEDLINE search strategy. **Appendix C.** Excluded studies. **Appendix D.** WinBUGS code. **Appendix E.** Similarity assessment. **Appendix F.** Results of the random-effects analyses. (DOCX 247 kb)

## Abbreviations

ABSSSI: Acute bacterial skin and skin structure infection
AE: Adverse event
CI: Confidence interval
CrI: Credible interval
cSSSI: Complicated skin and skin structure infections
cSSTI: Complicated skin and soft tissue infections
EOT: End of treatment
FDA: Food and Drug Administration
ITC: Indirect treatment comparison
ITT: Intention to treat
mITT: Modified intention to treat
MRSA: Methicillin-resistant *Staphylococcus aureus*
MSSA: Methicillin-sensitive *Staphylococcus aureus*
NICE: National Institute for Health and Care Excellence
NMA: Network meta-analysis
OR: Odds ratio
PBAC: Pharmaceutical Benefits Advisory Committee
PRISMA: Preferred Reporting Items for Systematic Reviews and Meta-Analyses
PTE: Post-treatment evaluation
RCT: Randomised controlled trial
TOC: Test of cure.
