# Supplementary material for: Systematic review and network meta-analysis of tedizolid for the treatment of acute bacterial skin and skin structure infections caused by MRSA
Source: BMC Infect Dis. 2017 Jan 7;17:39. doi: 10.1186/s12879-016-2100-3 (PMC5219662; doi:10.1186/s12879-016-2100-3)
Supplement: Additional file 1: Appendix A. — Eligibility criteria. Appendix B. MEDLINE search strategy. Appendix C. Excluded studies. Appendix D. WinBUGS code. Appendix E. Similarity assessment. Appendix F. Results of the random-effects analyses. (DOCX 247 kb) [file 12879_2016_2100_MOESM1_ESM.docx]

**SUPPLEMENTARY APPENDICES**

APPENDIX A Eligibility criteria

APPENDIX B MEDLINE search strategy

APPENDIX C Excluded studies

APPENDIX D WinBUGS code

Section D.1 NMA model

Section D.2 Input data for the models

APPENDIX E Similarity assessment

Section E.1 Assessment of similarity of trials for indirect comparison

Section E.2 Similarity assessment of common treatment arms

Section E.3 Definition of response as reported in the included trials

Section E.4 Analysis populations defined in the included trials

APPENDIX F Results of the random-effects analyses

**APPENDIX A Eligibility criteria**

**Protocol**

We will conduct the systematic literature review according to the principles of systematic reviewing as set out in the Centre for Reviews and Dissemination (CRD) [[11](#_ENREF_11)] and National Institute for Health and Care Excellence (NICE) [[12](#_ENREF_12)] guidance. To identify relevant evidence, a clear definition of the study participants, interventions, comparison groups, outcomes and study types of interest are required. These are described in detail next.

**Population**

Studies will be eligible for inclusion in the review if they assess adults with suspected or documented methicillin-resistant *Staphylococcus aureus* (MRSA)-associated acute bacterial skin and skin structure infection (ABSSSI) or complicated skin and skin structure infection (cSSSI).

ABSSSI is a relatively new classification introduced by the U.S. Food and Drug Administration (FDA) and is defined as *“a bacterial infection of the skin with a lesion size area of at least 75 cm^2^ (lesion size measured by the area of redness, edema, or induration)*” [[1](#_ENREF_1)]. We note that older studies will not use this new classification. Therefore, studies will be eligible for inclusion in the review if they report data for patients described as having ABSSSI (as described above) or any of the following infections that fall under the ABSSSI classification [[1](#_ENREF_1)]:

- Cellulitis/erysipelas: A diffuse skin infection characterized by spreading areas of redness, edema and/or induration;
- Major cutaneous abscess: An infection characterized by a collection of pus within the dermis or deeper that is accompanied by redness, edema and/or induration;
- Wound infection: An infection characterized by purulent drainage from a wound with surrounding redness, edema and/or induration.

Clinical input will be sought when alternative diagnosis criteria are reported in the literature.

A number of bacterial pathogens are associated with ABSSSI and often the specific pathogen is unknown when patients enter a trial. Studies in which ABSSSI is assumed, suspected or documented to be MRSA-associated will be eligible for inclusion. Studies assessing mixed populations (i.e. ABSSSI is found to be caused by a range of pathogens including MRSA) will be eligible for inclusion and, when possible, data will be collected for the MRSA-associated ABSSSI subgroup separately.

Studies that report data only for patients with ABSSSI *known* to be associated with pathogens other than MRSA will not be eligible for inclusion.

**Interventions**

Studies that evaluate tedizolid for the treatment of MRSA-associated ABSSSI will be eligible for inclusion in this review. Studies of the following interventions will also be eligible for inclusion with the intention of exploring options for network meta-analysis providing indirect comparisons to tedizolid:

- Vancomycin;
- Linezolid;
- Daptomycin;
- Teicoplanin;
- Tigecycline;
- Ceftaroline;
- Telavancin.

Studies of any treatment duration and any length of follow-up will be eligible for inclusion.

**Comparators**

Studies that compare any of the comparators of interest against any of the following will be eligible for inclusion in the NMA:

- Tedizolid;
- Vancomycin;
- Linezolid;
- Daptomycin;
- Teicoplanin;
- Tigecycline;
- Ceftaroline;
- Telavancin.

**Outcomes**

Studies will be eligible for inclusion if they assess one or more of the following outcomes:

- Response rate at test of cure^^[[1]](#footnote-1)^^ (TOC) or similar end point;
- Cure or improvement at 48–72 hours;
- Any serious adverse events (SAEs);
- SAEs leading to treatment discontinuation;
- Gastrointestinal AEs;
- Nephrotoxicity;
- Neurotoxicity (including optic neuropathy);
- Hepatotoxicity;
- Recurrence;
- Sustained response;
- Resistance.

When possible, data will also be collected for the following patient subgroups of interest:

- Obese patients (body mass index [BMI] ≥30 kg/m^2^);
- Patients with impaired renal function (serum creatinine levels >1.5 mg/dL);
- Patients taking concomitant selective serotonin reuptake inhibitors (SSRIs).

**Study design**

Randomized controlled trials of any size and duration, published as full reports, will be eligible for inclusion in the review and NMA.

Studies published as conference abstracts or presentations only will not be eligible for inclusion in the review and NMA because they are likely to provide insufficient information to permit an assessment of similarity and/or adequate outcome data.

Systematic reviews will be eligible for inclusion in the review as sources of references to primary studies.

**Limits**

No date or language limits will be applied to the searches.

Studies reported in languages other than English will not be extracted but will be listed in a table for information only.

# APPENDIX B MEDLINE search strategy

**MEDLINE(R) in-process & other non-indexed citations and MEDLINE(R) 1946 to present**

Interface / URL: OvidSP

Search date: 06/02/14

Retrieved records: **1936**

Search strategy:

1 Skin Diseases, Bacterial/ **2725**

2 exp Staphylococcal Skin Infections/ **4223**

3 Cellulitis/ **6179**

4 Erysipelas/ **1135**

5 Abscess/ **22255**

6 ((acute or complicated or complex) and (skin adj4 infect$)).ti,ab,kf. **1941**

7 ((acute or complicated or complex) and (soft tissue$ adj4 infect$)).ti,ab,kf. **1090**

8 ((acute or complicated or complex) and (connective tissue$ adj4 infect$)).ti,ab,kf. **55**

9 ((acute or complicated or complex) and (cutaneous adj4 infect$)).ti,ab,kf. **405**

10 ((acute or complicated or complex) and (wound$ adj4 infect$)).ti,ab,kf. **2993**

11 ((acute or complicated or complex) and (lacerat$ adj4 infect$)).ti,ab,kf. **15**

12 ((acute or complicated or complex) and (burn$1 adj4 infect$)).ti,ab,kf. **178**

13 ((acute or complicated or complex) and (surg$ adj4 infect$)).ti,ab,kf. **2302**

14 ((acute or complicated or complex) and (ulcer$ adj4 infect$)).ti,ab,kf. **485**

15 ((acute or complicated or complex) and (incision$ adj4 infect$)).ti,ab,kf. **101**

16 ((acute or complicated or complex) and ((bite or bites or biting) adj4 infect$)).ti,ab,kf. **181**

17 ((acute or complicated or complex) and (abscess$ adj4 infect$)).ti,ab,kf. **751**

18 (cellulitis or phlegmon or erysipelas).ti,ab,kf. **8781**

19 ((acute or complicated or complex) and (staphylococc$ or mrsa or mssa)).ti,ab,kf. **10053**

20 ((staphylococc$ or mrsa or mssa) adj4 infect$).ti,ab,kf. **16188**

21 (gram-positive adj4 infect$).ti,ab,kf. **2408**

22 ((skin or cutaneous or soft tissue$ or connective tissue$) adj4 abscess$).ti,ab,kf. **1347**

23 (csssi or csssis or absssi or absssis or ssti or sstis).ti,ab,kf. **810**

24 (complicated sssi or complicated sssis or acute bacterial sssi or acute bacterial sssis).ti,ab,kf. **14**

25 (skin structure$1 adj4 infect$).ti,ab,kf. **716**

26 or/1-25 **71877**

27 (torezolid or tedizolid or tr701 or tr-701 or tr700 or tr-700 or da7218 or da 7218 or da7157 or da 7157 or 856866-72-3 or "856866723" or 856867-55-5 or "856867555" or 97HLQ82NGL).ti,ab,kf,rn. **62**

28 Vancomycin/ **9941**

29 (vancomycin or vancomicin or vancomycine or 1404-90-6 or "1404906" or 1404-93-9 or "1404939" or 6Q205EH1VU).ti,ab,kf,rn. **19838**

30 (amplobac$ or balcorin$ or dhacocin$ or diatracin$ or edicin$ or icoplax$ or ifavac$ or kavocil$ or lyphocin$ or vagran$ or vanauras$ or vanbiotic$ or vancam$ or vanccostacin$ or vancep$ or vancin$ or vanco$1 or vancocid$ or vancocin$ or vancogen$ or vancoled$ or vancomax$ or vancomet$ or vancomicina$ or vancor$ or vancox$ or vancotex$ or vancozin$ or vanmicina$ or vanococin$ or vanosyn$ or vantocil$ or varedet$ or vidantin$ or voncon$).ti,ab,kf,rn. **307**

31 (linezolid or zyvox$ or linospan$ or arlin$1 or u100766 or u 100766 or pnu 100766 or pnu100766 or 165800-03-3 or "165800033" or ISQ9I6J12J).ti,ab,kf,rn. **3408**

32 Daptomycin/ **1198**

33 (daptomycin or daptomicin or deptomycin or cubicin$ or cidecin$ or dapcin$ or ly 146032 or ly146032 or 1030600-53-3 or "1030600533" or 103060-53-3 or "103060533" or NWQ5N31VKK).ti,ab,kf,rn. **1799**

34 Teicoplanin/ **1858**

35 (teicoplanin$ or teichomycin$ or teichomicin$ or teichoplanin$ or tagocid$ or targocid$ or targosid$ or 61036-62-2 or "61036622" or 61036-64-4 or "61036644" or 4U3D3YY81M).ti,ab,kf,rn. **3119**

36 (tigecyclin$ or tigeciclin$ or gar 936 or gar936 or tbgmino$ or tbg mino$ or tygacil$ or 220620-09-7 or "220620097" or 70JE2N95KR).ti,ab,kf,rn. **1563**

37 (ceftarolin$ or teflaro$ or zinforo$ or "ppi 0903" or ppi0903 or ppi 0903m or ppi0903m or "ppi 0903 m" or ppi0903 m or tak 599 or tak599 or t 91825 or t91825 or 189345-04-8 or 1134503-56-2 or 1210978-33-8 or 229016-73-3 or 400827-46-5 or 400827-55-6 or 400827-79-4 or 595568-96-0 or 866021-48-9 or "189345048" or "1134503562" or "1210978338" or "229016733" or "400827465" or "400827556" or "400827794" or "595568960" or "866021489" or H36Z0FHR8K).ti,ab,kf,rn. **225**

38 (telavancin$ or vibativ$ or arbelic$ or td 6424 or td6424 or 372151-71-8 or "372151718" or 380636-75-9 or "380636759" or 560130-42-9 or "560130429" or XK134822Z0).ti,ab,kf,rn. **245**

39 or/27-38 **24913**

40 26 and 39 **4164**

41 exp animals/ not humans/ **3869593**

42 (news or editorial or comment or case reports).pt. **2580622**

43 case report.ti. **153764**

44 40 not (41 or 42 or 43) **3170**

45 remove duplicates from 44 **3145**

46 randomized controlled trial.pt. **360585**

47 controlled clinical trial.pt. **87017**

48 randomi?ed.ab. **335708**

49 placebo.ab. **148912**

50 drug therapy.fs. **1653127**

51 randomly.ab. **204719**

52 trial.ab. **289920**

53 groups.ab. **1310023**

54 or/46-53 **3243181**

55 45 and 54 **1936**

# APPENDIX C Excluded studies

**Table B.1** List of excluded studies and reasons for exclusion, following full-text review

| Reference | Reason for exclusion |
| --- | --- |
| Abbanat D, Macielag M, Bush K. Novel antibacterial agents for the treatment of serious Gram-positive infections. Expert Opin Investig Drugs. 2003;12:379-99. | Not an SR (no methods reported) |
| Aguado JM, Torres A, Munoz P, Soriano A, Carratala J, Guirao X, et al. Severe, non-bacteremic infections in ICU patients. Enferm Infecc Microbiol Clin. 2011;29 Suppl 4:1-9. | Non-SR and commentary on a selection of papers focusing on pneumonia diagnosis |
| Aguilar L, Barberan J, Prieto J, Gimenez M. [Improvement of bactericide activity in hospital treatment of gram positive infections]. Rev Esp Quimioter. 2008;21:37-44. | Spanish; general review of factors affecting the activity of bactericidal drugs in hospital treatment of gram-positive infections |
| Aksoy DY, Unal S. New antimicrobial agents for the treatment of Gram-positive bacterial infections. Clin Microbiol Infect. 2008;14:411-20. | General review of pharmacology and efficacy of antimicrobials for gram-positive bacterial infections |
| Alberghina A, Romeo B, Pessione E, Mauri A, Enrichens F, Sciascia C, et al. [Efficacy and tolerance of teicoplanin in postoperative infections caused by gram-positive microorganisms]. Minerva Chir. 1987;42:1339-45. | Not an RCT |
| Anstead GM, Owens AD. Recent advances in the treatment of infections due to resistant Staphylococcus aureus. Curr Opin Infect Dis. 2004;17:549-55. | Not an SR (no methods reported) |
| Anstead GM, Quinones-Nazario G, Lewis JS 2nd. Treatment of infections caused by resistant Staphylococcus aureus. Methods Mol Biol. 2007;391:227-58. | Not an SR (no methods reported) |
| Appelbaum PC, Jacobs MR. Recently approved and investigational antibiotics for treatment of severe infections caused by Gram-positive bacteria. Curr Opin Microbiol. 2005;8:510-7. | General review of the effectiveness of antibiotic treatments for gram-positive bacterial infections |
| Arbeit RD, Maki D, Tally FP, Campanaro E, Eisenstein BI; Daptomycin 98-01 and 99-01 Investigators. The safety and efficacy of daptomycin for the treatment of complicated skin and skin-structure infections. Clin Infect Dis. 2004;38:1673-81. | Combined results from 2 RCTs (Cubist) |
| Attwood RJ, LaPlante KL. Telavancin: a novel lipoglycopeptide antimicrobial agent. Am J Health SystPharm. 2007;64:2335-48. | General review of pharmacology, activity, pharmacokinetics, pharmacodynamics, clinical efficacy, safety, dosage and place in therapy of telavancin |
| Auwaerter PG. Cellulitis, skin abscesses, and community-acquired methicillin-resistant Staphylococcus aureus. Adv Studies Med. 2006;6:62-70. | General review of clinical features and treatment of common SSTIs, *Staphylococcus aureus* antibiotic resistance and community-acquired MRSA |
| Avdic E, Cosgrove SE. Management and control strategies for community-associated methicillin-resistant Staphylococcus aureus. Expert Opin Pharmacother. 2008;9:1463-79. | Not an SR (searched MEDLINE but no other methods reported) |
| Backx M, Healy B. Serious staphylococcal infections. Clin Med. 2008;8:535-8. | General article on diagnosis and management of diseases caused by staphylococcus infections |
| Bain KT, Wittbrodt ET. Linezolid for the treatment of resistant gram-positive cocci. Ann Pharmacother. 2001;35:566-75. | Non-SR looking at therapeutic issues and pharmacology, efficacy, safety, dosing and pharmacoeconomics of linezolid for gram-positive bacterial infections |
| Bal AM, Gould IM. Antibiotic resistance in Staphylococcus aureus and its relevance in therapy. Expert Opin Pharmacother. 2005;6:2257-69. | Not an SR (no methods reported) |
| Baltz RH, Miao V, Wrigley SK. Natural products to drugs: daptomycin and related lipopeptide antibiotics. Nat Prod Rep. 2005;22:717-41. | General review of lipopeptide antibiotics up to 2005: development, structure, reaction mechanisms, biosynthesis and modification |
| Bamberger DM, Boyd SE. Management of Staphylococcus aureus infections. Am Fam Physician. 2005;72:2474-81. | General review of costs and management of *S. aureus* in skin, soft-tissue, catheter-related, bone, joint, pulmonary and central nervous system infections |
| Banwan K, Senok AC, Rotimi VO. Antibiotic therapeutic options for infections caused by drug-resistant Gram-positive cocci. J Infect Public Health. 2009;2:62-73. | Non-SR of pharmacology, efficacy and safety of antibiotics (including daptomycin, tigecycline and linezolid) for gram-positive resistant bacterial infections |
| Barriere SL. ATLAS trials: efficacy and safety of telavancin compared with vancomycin for the treatment of skin infections. Future Microbiol. 2010;5:1765-73. | Not an RCT or SR; narrative review of ATLAS trial |
| Bassetti M, Giacobbe DR, Taramasso L. Tigecycline use in hospital and its potential role in infection control. Eur Infect Dis. 2012;6:57-60. | Review article summarizing the current position on the use of tigecycline in the treatment of cSSSIs, cIAIs, ventilator-associated pneumonia and *Clostridium difficile* and the potential role of this drug in infection control |
| Bassetti M, Mikulska M, Righi E, Nicolini L, Viscoli C. The role of telavancin in the treatment of MRSA infections in hospital. Expert Opin Investig Drugs. 2009;18:521-9. | Drug evaluation reviewing the microbiological activity, pharmacology, efficacy and safety of telavancin |
| Batts DH. Linezolid--a new option for treating gram-positive infections. Oncology (Williston Park). 2000;14 Suppl 6:23-9. | Not an SR (no methods reported) |
| Bazan JA, Martin SI. Ceftaroline fosamil: a novel broad-spectrum cephalosporin. Drugs Today. 2010;46:743-55. | Not an SR (no methods reported) |
| Bazan JA, Martin SI, Kaye KM. Newer beta-lactam antibiotics: doripenem, ceftobiprole, ceftaroline and cefepime. Med Clin North Am. 2011;95:743-60, viii. | General review of beta-lactam antibiotics |
| Bazan JA, Martin SI, Kaye KM. Newer beta-lactam antibiotics: doripenem, ceftobiprole, ceftaroline, and cefepime. Infect Dis Clin North Am. 2009;23:983-96. | Not an SR (no methods reported) |
| Beiras-Fernandez A, Vogt F, Sodian R, Weis F. Daptomycin: a novel lipopeptide antibiotic against Gram-positive pathogens. Infect Drug Resist. 2010;3:95-101. | General review of daptomycin for gram-positive pathogens |
| Belavic JM. Ceftaroline (Teflaro): a new cephalosporin. Nurse Pract. 2011;36:12-3. | News article on ceftaroline: dosing and administration, adverse reactions and warnings/precautions |
| Bencke A, Heineck I. [Efficacy and safety of tigecycline, the first antibiotic of the glycylcyclines's class.] Latin Am J Pharm. 2008;27:928-37. | Unclear whether an SR because has search strategy, but published in Portuguese or Spanish |
| Biek D, Critchley IA, Riccobene TA, Thye DA. Ceftaroline fosamil: a novel broad-spectrum cephalosporin with expanded anti-Gram-positive activity. J Antimicrob Chemother. 2010;65 Suppl 4:iv9-16. | Non-SR of pharmacology and clinical efficacy of ceftaroline |
| Bosso JA. The antimicrobial armamentarium: evaluating current and future treatment options. Pharmacotherapy. 2005;25:55S-62S. | Not an SR (no methods reported) |
| Bounthavong M, Hsu DI. Efficacy and safety of linezolid in methicillin-resistant Staphylococcus aureus (MRSA) complicated skin and soft tissue infection (cSSTI): a meta-analysis. Curr Med Res Opin. 2010;26:407-21. | DARE abstract: primary publication has been retrieved |
| Bounthavong M, Hsu DI, Okamoto MP. Cost-effectiveness analysis of linezolid vs. vancomycin in treating methicillin-resistant Staphylococcus aureus complicated skin and soft tissue infections using a decision analytic model. Int J Clin Pract. 2009;63:376-86. | Cost-effectiveness analysis of treatments for MRSA cSSTIs |
| Bounthavong M, Zargarzadeh A, Hsu DI, Vanness DJ. Cost-effectiveness analysis of linezolid, daptomycin, and vancomycin in methicillin-resistant Staphylococcus aureus: complicated skin and skin structure infection using Bayesian methods for evidence synthesis. Value Health. 2011;14:631-9. | Cost-effectiveness analysis of treatments for MRSA cSSSI |
| Bouza E, Munoz P. Linezolid: pharmacokinetic characteristics and clinical studies. Clin Microbiol Infect. 2001;7 Suppl 4:75-82. | Non-SR of pharmacology and clinical study evidence for linezolid used to treat multi-resistant gram-positive infections and different indications |
| Brickner SJ, Barbachyn MR, Hutchinson DK, Manninen PR. Linezolid (ZYVOX), the first member of a completely new class of antibacterial agents for treatment of serious gram-positive infections. J Med Chem. 2008;51:1981-90. | Award address; overview of the discovery and evaluation of linezolid, as carried out by the award recipients |
| Brink AJ, Bizos D, Boffard KD, Feldman C, Grolman DC, Pretorius J, et al. Guideline: appropriate use of tigecycline. S Afr Med J. 2010;100:388-94. | Guideline recommendations on use of tigecycline, drawn up from multidisciplinary meeting of relevant South African associations/bodies. No evident systematic approach to identifying evidence |
| Brogden RN, Peters DH. Teicoplanin - a reappraisal of its antimicrobial activity, pharmacokinetic properties and therapeutic efficacy. Drugs. 1994;47:823-54. | Update of previous review with data relating to in vitro activity, pharmacokinetic profile, clinical efficacy and tolerability of teicoplanin |
| Bryskier A. Anti-MRSA agents: under investigation, in the exploratory phase and clinically available. Expert Rev Anti Infect Ther. 2005;3:505-53. | Not an SR (no methods reported) |
| Butterfield JM, Lawrence KR, Reisman A, Huang DB, Thompson CA, Lodise TP. Comparison of serotonin toxicity with concomitant use of either linezolid or comparators and serotonergic agents: an analysis of Phase III and IV randomized clinical trial data. J Antimicrob Chemother. 2012;67:494-502. | A review/analysis (not an SR or meta-analysis) of AEs in clinical studies of linezolid for a variety of indications, to evaluate serotonin toxicity |
| Cafferkey MT, Hone R, Keane CT. Severe staphylococcal infections treated with vancomycin. J Antimicrob Chemother. 1982;9:69-74. | Not an RCT/SR (single-arm case series) |
| Carpenter CF, Chambers HF. Daptomycin: another novel agent for treating infections due to drug-resistant gram-positive pathogens. Clin Infect Dis. 2004;38:994-1000. | General review of activity, pharmacology, efficacy and safety of daptomycin in drug-resistant gram-positive infections |
| Cattoir V, Daurel C. [Update on antimicrobial chemotherapy]. Med Mal Infect. 2010;40:135-54. | General review of microbiological, pharmacological and clinical properties of systemic antibiotics marketed in France, those in development and those available in other countries |
| Ceftaroline fosamil (Teflaro) - a new IV cephalosporin. Med Lett Drugs Ther. 2011;53:5-6. | Drug evaluation of newly FDA-approved drug (ceftaroline) |
| Cepeda JA, Whitehouse T, Cooper B, Hails J, Jones K, Kwaku F, et al. Linezolid versus teicoplanin in the treatment of Gram-positive infections in the critically ill: a randomized, double-blind, multicentre study. J Antimicrob Chemother. 2004;53:345-55. | Critically ill patients with gram-positive infections, including SSTIs; no data specifically relating to patients with SSTIs |
| Chambers HF, Hegde SS. Combating the growing problem of methicillin-resistant Staphylococcus aureus: do the newer antibiotics represent a better alternative to vancomycin? Expert Rev Anti Infect Ther. 2007;5:333-5. | Editorial discussing the problem of MRSA and alternative treatments to vancomycin |
| Chambers HF. Treatment of infection and colonization caused by methicillin-resistant Staphylococcus aureus. Infect Control Hosp Epidemiol. 1991;12:29-35. | Not an SR (no methods reported) |
| Charbonneau P, Harding I, Garaud JJ, Aubertin J, Brunet F, Domart Y. Teicoplanin: a well-tolerated and easily administered alternative to vancomycin for gram-positive infections in intensive care patients. Intensive Care Med. 1994;20 Suppl 4:S35-42. | Both intervention and comparator groups received concomitant netilmicin |
| Charneski L, Patel PN, Sym D. Telavancin: a novel lipoglycopeptide antibiotic. Ann Pharmacother. 2009;43:928-38. | Not an SR (has search strategy but no other review methods reported) |
| Chua K, Howden BP. Treating Gram-positive infections: vancomycin update and the whys, wherefores and evidence base for continuous infusion of anti-Gram-positive antibiotics. Curr Opin Infect Dis. 2009;22:525-34. | General review of studies relating to the clinical use of vancomycin and the role of continuous infusion antibiotics in treating gram-positive infections, focusing on *S. aureus* |
| Coll PP, Nurse BA. Implications of methicillin-resistant Staphylococcus aureus on nursing home practice. J Am Board Fam Pract. 1992;5:193-200. | Not an SR (has search but no other methodology) |
| Cook FV, Farrar WE Jr. Vancomycin revisited. Ann Intern Med. 1978;88:813-8. | Not an SR (no methods reported) |
| Cooke FJ, Brown NM. Community-associated methicillin-resistant Staphylococcus aureus infections. Br Med Bull. 2010;94:215-27. | General review of the fundamentals of community-acquired MRSA infections: epidemiology, prevalence, clinical presentation, molecular typing, diagnosis etc., but not efficacy/safety |
| Corey GR, Stryjewski ME, Fowler G, Teglia O, Hopkins A, Kitt M, et al. ATLAS 2: a double-blind, randomised, active controlled, multinational Phase 3 study comparing telavancin with vancomycin for the treatment of patients with complicated skin and skin structure infections. Int J Antimicrob Agents. 2007;29:S217. | Conference abstract |
| Corey GR, Stryjewski ME, Fowler VG, Lentnek A, Hopkins A, Kitt MM, et al. Baseline characteristics of patients, with or without MRSA, in two double-blind, randomised, multinational, Phase 3 studies comparing telavancin with vancomycin for the treatment of complicated skin and skin structure infections. Int J Antimicrob Agents. 2007;29:S29. | Conference abstract |
| Corey GR, Stryjewski ME, Fowler VG, Skerk V, Hopkins A, Kitt MM, et al. The ATLAS studies: double-blind, randomised, active controlled, multinational Phase 3 trials comparing telavancin with vancomycin for the treatment of complicated skin and skin structure infections. Int J Antimicrob Agents. 2007;29:S215-6. | Conference abstract |
| Corey GR, Stryjewski ME, Weyenberg W, Yasothan U, Kirkpatrick P. Telavancin. Nat Rev Drug Disc. 2009;8:929-30. | General overview of telavancin: discovery, drug properties, clinical data and indications |
| Cornaglia G, Rossolini GM. Forthcoming therapeutic perspectives for infections due to multidrug-resistant Gram-positive pathogens. Clin Microbiol Infect. 2009;15:218-23. | Overview of antibiotic resistance and new drug agents, based on conference material and current literature at the time |
| Corti G, Cinelli R, Paradisi F. Clinical and microbiologic efficacy and safety profile of linezolid, a new oxazolidinone antibiotic. Int J Antimicrob Agents. 2000;16:527-30. | General review of bacteriological and pharmacokinetic properties and efficacy and safety of linezolid |
| Cunha BA, Ristuccia AM. Clinical usefulness of vancomycin. Clin Pharm. 1983;2:417-24. | Not an SR (no methods reported) |
| Cunha BA. Methicillin-resistant Staphylococcus aureus: clinical manifestations and antimicrobial therapy. Clin Microbiol Infect. 2005;11 Suppl 4:33-42. | Not an SR (no methods reported) |
| Curcio D. Resistant pathogen-associated skin and skin-structure infections: antibiotic options. Expert Rev Anti Infect Ther. 2010;8:1019-36. | Calls itself an evidence-based review but no methods reported; reviews multidrug-resistant pathogens causing cSSSIs, implications of resistance and new therapeutic options |
| Das D, Tulkens PM, Mehra P, Fang E, Prokocimer P. Tedizolid phosphate for the management of acute bacterial skin and skin structure infections: safety summary. Clin Infect Dis. 2014;58 Suppl 1:S51-7. | Not an SR |
| De Cock E, Sorensen S, Levrat F, Besnier JM, Dupon M, Guery B, et al. Cost-effectiveness of linezolid versus vancomycin for hospitalized patients with complicated skin and soft-tissue infections in France. Med Mal Infect. 2009;39:330-40. | Cost-effectiveness analysis of treatments for MRSA-associated cSSTIs |
| De Rosa GG. [Infections in the immunocompromised host: role of daptomycin]. Infez Med. 2009;17 Suppl 3:25-31. | Italian; general review plus case reports relating to daptomycin use in immunocompromised patients |
| Deresinski S. Vancomycin: does it still have a role as an antistaphylococcal agent? Expert Rev Anti Infect Ther. 2007;5:393-401. | Not an SR |
| Diekema DJ, Jones RN. Oxazolidinone antibiotics. Lancet. 2001;358:1975-82. | General review of action, resistance, pharmacology, efficacy and safety of oxazolidinone antibiotics in treatment of gram-positive pathogens |
| Doan T-L, Fung HB, Mehta D, Riska PF. Tigecycline: a glycylcycline antimicrobial agent. Clin Ther. 2006;28:1079-106. | Non-SR of pharmacology, efficacy and safety of tigecycline in cSSSIs and cIAIs |
| Dominguez EA. Single-agent therapy with tigecycline in the treatment of complicated skin and skin structure and complicated intraabdominal infections. Infect Dis Clin Pract. 2009;17:144-9. | Not an SR (no methods reported) |
| Drugs for staphylococcal infections. Med Lett Drugs Ther. 1973;15:93-4. | Not an SR |
| Duane TM, Capitano B, Puzniak LA, Biswas P, Joshi M. The impact of linezolid versus vancomycin on surgical interventions for complicated skin and skin structure infections caused by methicillin-resistant Staphylococcus aureus. Surg Infect (Larchmnt). 2013;14:401-7. | Conference abstract |
| Duane TM, Weigelt JA, Puzniak LA, Huang DB. Linezolid and vancomycin in treatment of lower-extremity complicated skin and skin structure infections caused by methicillin-resistant Staphylococcus aureus in patients with and without vascular disease. Surg Infect (Larchmnt). 2012;13:147-53. | Not an SR or RCT; pooled data from 2 RCTs |
| Dunbar LM, Tang DM, Manausa RM. A review of telavancin in the treatment of complicated skin and skin structure infections (cSSSI). Ther Clin Risk Manag. 2008;4:235-44. | Non-SR of telavancin in treatment of cSSSI |
| Dutronc H, Bocquentin F, Galperine T, Lafarie-Castet S, Dupon M. [Linezolid, the first oxazolidinone antibiotic]. Med Mal Infect. 2005;35:427-34. | French; general review of microbiology and pharmacology of linezolid in gram-positive infections such as SSTIs |
| Eckmann C, Dryden M. Treatment of complicated skin and soft-tissue infections caused by resistant bacteria: value of linezolid, tigecycline, daptomycin and vancomycin. Eur J Med Res. 2010;15:554-63. | General review of linezolid, tigecycline, daptomycin and vancomycin for cSSTI caused by resistant bacteria |
| Eggleston M, Ofosu J. Teicoplanin--a new agent for gram-positive bacterial infections. Infect Control Hosp Epidemiol. 1988;9:209-11. | Not an SR (no methods reported) |
| Eisenstein BI. Lipopeptides, focusing on daptomycin, for the treatment of Gram-positive infections. Expert Opin Investig Drugs. 2004;13:1159-69. | Not an SR (no methods reported) |
| Eisenstein BI. Treatment of staphylococcal infections with cyclic lipopeptides. Clin Microbiol Infect. 2008;14 Suppl 2:10-6. | General review of daptomycin for treating gram-positive infections, particularly SSTIs and SAB and infective endocarditis |
| Eleftheriadou I, Tentolouris N, Argiana V, Jude E, Boulton AJ. Methicillin-resistant Staphylococcus aureus in diabetic foot infections. Drugs. 2010;70:1785-97. | Not an SR (no methods reported) |
| Ellis MW, Lewis JS 2nd. Treatment approaches for community-acquired methicillin-resistant Staphylococcus aureus infections. Curr Opin Infect Dis. 2005;18:496-501. | Not an SR (no methodology) |
| Falagas ME, Vardakas KZ. Benefit-risk assessment of linezolid for serious gram-positive bacterial infections. Drug Saf. 2008;31:753-68. | Risk-benefit assessment; non-systematic overview of evidence from RCTs in specific populations, including MRSA SSTIs |
| Fenton C, Keating GM, Curran MP. Daptomycin. Drugs. 2004;64:445-55; discussion 457-8. | Not an SR (no methods reported) |
| Fierlbeck G, Duvall D, Bruss J, Molinari M, Winterhalter B, Grassi C. No increased risk of anemia in the treatment of Gram-positive infections with linezolid: results of 7 multinational comparator-controlled clinical trials. Ann Oncol. 2000;11:150. | Conference abstract |
| Fraise AP. Tigecycline: the answer to beta-lactam and fluoroquinolone resistance? J Infect. 2006;53:293-300. | General review of antibiotic resistance and drug development, in particular tigecycline |
| Fry DE, Barie PS. The changing face of Staphylococcus aureus: a continuing surgical challenge. Surg Infect (Larchmnt). 2011;12:191-203. | Not an SR (no methods reported) |
| Fung HB, Kirschenbaum HL, Ojofeitimi BO. Linezolid: an oxazolidinone antimicrobial agent. Clin Ther. 2001;23:356-91. | Not an SR (has search but no other methods reported) |
| Garau J. Management of cSSTIs: the role of daptomycin. Curr Med Res Opin. 2006;22:2079-87. | Not an SR (authors state this was not intended as an SR) |
| Garnacho-Montero J, Amaya-Villar R, Gomez-Grande ML, Jerez V, Lorente-Ramos L, Loza A, et al. [Role of daptomycin in the empirical and directed therapy of infections caused by Gram-positive bacteria in the critically ill patient]. Rev Esp Quimioter. 2011;24:13-24. | Spanish; unclear whether this is an SR |
| Garrison MW, Kawamura NM, Wen MM. Ceftaroline fosamil: a new cephalosporin active against resistant Gram-positive organisms including MRSA. Expert Rev Anti Infect Ther. 2012;10:1087-103. | Not an SR/RCT |
| Garrison MW, Neumiller JJ, Setter SM. Tigecycline: an investigational glycylcycline antimicrobial with activity against resistant gram-positive organisms. Clin Ther. 2005;27:12-22. | General review of microbial resistance, pharmacology, activity and safety of tigecycline |
| Gennery BA, Cooper GL. Therapeutic approach to methicillin-resistant Staphylococcus aureus (MRSA) infections: vancomycin update. Br J Clin Pract Suppl. 1988;57:100-1. | Not an SR (no methods reported) |
| Ghasri P, Scheinfeld NS. Vibativ (telavancin) for complicated skin and skin structure infections. Skinmed. 2010;8:175-6. | News feature highlighting ”new” drug: Vibativ (telavancin) |
| Giamarellou H, Poulakou G. Pharmacokinetic and pharmacodynamic evaluation of tigecycline. Expert Opin Drug Metab Toxicol. 2011;7:1459-70. | Review/opinion article highlighting areas in which knowledge of pharmacokinetics/pharmacodynamics of tigecycline has been strengthened and questioned |
| Glick HA, Willke R, Rittenhouse BE, Balan DA, Orzol SM, Li JZ. Comparing hospital costs between linezolid and vancomycin in the treatment of methicillin-resistant staphylococcal species (MRSS) infections: a randomized multi-center clinical trial. Value Health. 2002;5:565-6. | Conference abstract |
| Glinka TW. Novel cephalosporins for the treatment of MRSA infections. Curr Opin Investig Drugs. 2002;3:206-17. | Not an SR (no methods reported) |
| Goodman JJ, Martin SI. Critical appraisal of ceftaroline in the management of community-acquired bacterial pneumonia and skin infections. Ther Clin Risk Manag. 2012;8:149-56. | Non-SR of clinical efficacy in ABSSSI and CABP, microbiology, pharmacokinetic and safety of ceftaroline |
| Gould FK. Linezolid: safety and efficacy in special populations. J Antimicrob Chemother. 2011;66 Suppl 4:iv3-6. | Not an SR (has search strategy but no other methods were reported) |
| Gould IM, David MZ, Esposito S, Garau J, Lina G, Mazzei T, et al. New insights into meticillin-resistant Staphylococcus aureus (MRSA) pathogenesis, treatment and resistance. Int J Antimicrob Agents. 2012;39:96-104. | General review covering pathogenesis, treatment and resistance of MRSA, based on discussions at an expert panel meeting |
| Graham RC Jr. Antibiotics for treatment of infections caused by gram-positive cocci. Med Clin North Am. 1974;58:505-17. | Not an SR |
| Grau S, Aguado JM, Mateu-de Antonio J, Gonzalez P, Del Castillo A. Economic evaluation of linezolid versus teicoplanin for the treatment of infections caused by gram-positive microorganisms in Spain. J Chemother. 2007;19:398-409. | Economic evaluation |
| Griffith RS. Introduction to vancomycin. Rev Infect Diss. 1981;3 Suppl:S200-4. | Not an SR |
| BMJ Group. What role for tigecycline in infections? Drug Ther Bull. 2008;46:62-4. | Non-SR of efficacy and cost-effectiveness of tigecycline in SSTIs and IAIs |
| Gu B, Kelesidis T, Tsiodras S, Hindler J, Humphries RM. The emerging problem of linezolid-resistant Staphylococcus. J Antimicrob Chemother. 2013;68:4-11. | Not MRSA: SR of reports of linezolid-resistant *Staphylococcus* |
| Guay DR. Treatment of bacterial skin and skin structure infections. Expert Opin Pharmacother. 2003;4:1259-75. | Not an SR (no methods reported) |
| Guo Z, Lin Z, Huang P, Chen Q. Linezolid versus glycopeptides in the treatment of complicated skin and soft tissue infections: a meta-analysis of randomized controlled trials. Chin J Infect Chemother. 2011;11:268. | Conference abstract |
| Guskey MT, Tsuji BT. A comparative review of the lipoglycopeptides: oritavancin, dalbavancin, and telavancin. Pharmacotherapy. 2010;30:80-94. | General review comparing pharmacology of lipoglycopeptides, including telavancin |
| Hagihara M, Umemura T, Mori T, Mikamo H. Daptomycin approved in Japan for the treatment of methicillin-resistant Staphylococcus aureus. Ther Clin Risk Manag. 2012;8:79-86. | Non-SR article of daptomycin for MRSA |
| Hal SJ, Lodise TP, Paterson DL. The clinical significance of vancomycin minimum inhibitory concentration in Staphylococcus aureus infections: a systematic review and meta-analysis. Clin Infect Dis. 2012;54:755-71. | SR did not provide specific details of infections studied in the included studies (only whether bloodstream infection) |
| Hashisaki PA, Jacobson JA. Characteristics, control, and treatment of methicillin-resistant Staphylococcus aureus infections. Clin Pharm. 1982;1:343-8. | Not an SR (no methods reported) |
| Hau T. Efficacy and safety of linezolid in the treatment of skin and soft tissue infections. Eur J Clin Microbiol Infect Dis. 2002;21:491-8. | Non-SR of clinical trials of linezolid in SSTIs, pharmacoeconomic analysis and safety |
| Hernandez Marti V, Roma Sanchez E, Salavert Lleti M, Boso Ribelles V, Poveda Andres JL. [Daptomycin: revitalizing a former drug due to the need of new active agents against grampositive multiresistant bacterias]. Rev Esp Quimioter. 2007;20:261-76. | Non-SR of pharmacology, efficacy and safety of daptomycin in cSSTIs and bacteremia |
| Herrmann DJ, Peppard WJ, Ledeboer NA, Theesfeld ML, Weigelt JA, Buechel BJ. Linezolid for the treatment of drug-resistant infections. Expert Rev Anti Infect Ther. 2008;6:825-48. | Drug profile on linezolid: activity, pharmacology, on- and off-label clinical trial data, pharmacoeconomics and safety; also commentary and 5-year view of antimicrobial field in MRSA treatment |
| Huang DB, Reisman A, Hogan P. Clinical outcomes by methicillin-resistant Staphylococcus aureus staphylococcal cassette chromosome mec type: isolates recovered from a phase IV clinical trial of linezolid and vancomycin for complicated skin and skin structure infections. Antimicrob Agents Chemother. 2010;54:4036-7. | Letter reporting clinical and microbiological outcomes according to MRSA type in cSSSIs |
| Hylands J. Tigecycline: a new antibiotic. Intensive Crit Care Nurs. 2008;24:260-3. | Non-SR of dosing, clinical efficacy and side effects of tigecycline in SSTIs and IAIs |
| Ippolito G, Leone S, Lauria FN, Nicastri E, Wenzel RP. Methicillin-resistant Staphylococcus aureus: the superbug. Int J Infect Dis. 2010;14 Suppl 4:S7-11. | General review of the epidemiology, costs, outcome and treatment options for MRSA infections |
| Jacob JT, DiazGranados CA. High vancomycin minimum inhibitory concentration and clinical outcomes in adults with methicillin-resistant Staphylococcus aureus infections: a meta-analysis. Int J Infect Dis. 2013;17:e93-100. | SR but no relevant RCTs identified |
| Jafari Saraf L, Wilson SE. Telavancin, a new lipoglycopeptide antimicrobial, in complicated skin and soft tissue infections. Infect Drug Resist. 2011;4:87-95. | Non-SR article of telavancin for SSTIs |
| Jankowski A, Stefanik W. [Linezolid--novel antibiotic for the treatment of gram-positive bacterial infections]. Wiad Lek. 2006;59:727-31. | Unclear whether SR; no mention of SR methodology in sparse abstract; no tables of included studies |
| Jeu L, Fung HB. Daptomycin: a cyclic lipopeptide antimicrobial agent. Clin Ther. 2004;26:1728-57. | Not an SR (has search but no other methods reported) |
| Johnson MD, Decker CF. Antimicrobial agents in treatment of MRSA infections. Dis Mon. 2008;54:793-800. | General review of treatments for MRSA infections |
| Johnson SW, Drew RH, May DB. How long to treat with antibiotics following amputation in patients with diabetic foot infections? Are the 2012 IDSA DFI guidelines reasonable? J Clin Pharm Ther. 2013;38:85-8. | Commentary on antibiotic treatment guidelines |
| Juthani V, Zoumalan CI, Lisman RD, Rizk SS. Successful management of methicillin-resistant Staphylococcus aureus orbital cellulitis after blepharoplasty. PlastReconstr Surg. 2010;126:305e-7e. | Viewpoint on procedural complications/case report |
| Kanafani ZA, Corey GR. Ceftaroline: a cephalosporin with expanded Gram-positive activity. Future Microbiol. 2009;4:25-33. | Drug evaluation of ceftaroline: chemistry, microbiology, pharmacology, efficacy and safety |
| Kanafani ZA, Corey GR. Daptomycin: a rapidly bactericidal lipopeptide for the treatment of Gram-positive infections. Expert Rev Anti Infect Ther. 2007;5:177-84. | Not an SR (no methods reported) |
| Kanafani ZA. Ceftaroline fosamil: drug profile and clinical data. Future Microbiol. 2011;6:9-18. | Drug evaluation of newly FDA-approved drug (ceftaroline) |
| Kasbekar N. Tigecycline: a new glycylcycline antimicrobial agent. Am J Health Syst Pharm. 2006;63:1235-43. | Not an SR ( no methods reported) |
| Katz DE, Lindfield KC, Steenbergen JN, Benziger DP, Blackerby KJ, Knapp AG, et al. A pilot study of high-dose short duration daptomycin for the treatment of patients with complicated skin and skin structure infections caused by gram-positive bacteria. Int J Clin Pract. 2008;62:1455-64. | Comparator is vancomycin or semi-synthetic penicillin; results not presented separately for those receiving vancomycin |
| Kaushik D, Rathi S, Jain A. Ceftaroline: a comprehensive update. Int J Antimicrob Agents. 2011;37:389-95. | Non-SR of properties and action of ceftaroline |
| Kelesidis T. Safety and efficacy of daptomycin in the treatment of gram-positive pathogens. Clin Med Insights Ther. 2011;3:403-14. | Non-SR of the activity, pharmacokinetics, safety and efficacy of daptomycin for various indications, including cSSTIs, and at high doses |
| Kern WV. Daptomycin: first in a new class of antibiotics for complicated skin and soft-tissue infections. Int J Clin Pract. 2006;60:370-8. | General review of pharmacology, efficacy and safety of daptomycin in gram-positive bacterial infections, in particular cSSSIs |
| Khare M, Keady D. Antimicrobial therapy of methicillin resistant Staphylococcus aureus infection. Expert Opin Pharmacother. 2003;4:165-77. | Not an SR (no methods reported) |
| Khawcharoenporn T, Alan T. Oral antibiotic treatment for methicillin-resistant Staphylococcus aureus skin and soft tissue infections: review of the literature. Hawaii Med J. 2006;65:290-3. | Not an SR (has search strategy but no other methods were reported) |
| Kil EH, Heymann WR, Weinberg JM. Methicillin-resistant Staphylococcus aureus: an update for the dermatologist, part 3: clinical management. Cutis. 2008;81:327-35. | Continuing medical education article on the clinical management of MRSA infections |
| Kinney KK. Treatment of infections caused by antimicrobial-resistant gram-positive bacteria. Am J Med Sci. 2010;340:209-17. | Symposium article on treatment of drug-resistant gram-positive bacterial infections, focusing on MRSA |
| Kirby WM. Vancomycin therapy of severe staphylococcal infections. J Antimicrob Chemother. 1984;14 Suppl D:73-8. | General review of use of vancomycin, alone or with other drugs, in treatment of staphylococcal infections |
| Koenig MG. Staphylococcal infections--treatment and control. Dis Mon. 1968:Apr;1-36. | Not an SR |
| Kollef MH. New antimicrobial agents for methicillin-resistant Staphylococcus aureus. Crit Care Resusc. 2009;11:282-6. | General review of antibacterial agents that were in late stage of clinical development that showed potential for treatment of MRSA infections |
| Koning S, van der Sande R, Verhagen AP, van Suijlekom-Smit LW, Morris AD, Butler CC, et al. Interventions for impetigo. Cochrane Database Syst Rev. 2012;1:CD003261. | Cochrane review of RTCs of impetigo, not specifically MRSA |
| Konychev A, Heep M, Moritz RK, Kreuter A, Shulutko A, Fierlbeck G. A comparative randomised clinical trial against semisynthetic penicillins and glycopeptides supports the use of daptomycin as first-line treatment of complicated skin and soft-tissue infections in the elderly. Clin Microbiol Infect. 2012;18(suppl S3):838-9. | Conference abstract |
| Konychev A, Heep M, Moritz RKC, Kreuter A, Shulutko A, Fierlbeck G, et al. Safety and efficacy of daptomycin as first-line treatment for complicated skin and soft tissue infections in elderly patients: an open-label, multicentre, randomized phase IIIb trial. Drugs Aging. 2013;30:829-36. | Patients with gram-positive cSSTIs; results not reported according to pathogen (although vancomycin-treated patients have known/suspected MRSA) |
| Kosmidis C, Levine DP. Daptomycin: pharmacology and clinical use. Expert Opin Pharmacother. 2010;11:615-25. | General review/opinion article discussing pharmacology, resistance and clinical applications of daptomycin |
| Krause KM, Barriere SL, Kitt MM, Benton BM. In vitro activity of telavancin against Gram-positive isolates from complicated skin and skin structure infections: results from 2 phase 3 (ATLAS) clinical studies. Diagn Microbiol Infect Dis. 2010;68:181-5. | Types and distribution of bacterial species in isolates from cSSSIs (from patients in ATLAS clinical trials) |
| Kresken M. [How effective are linezolid and telithromycin? The resistance situation of pathogenic gram positive infections in Germany]. Pharm Unserer Zeit. 2004;33:20-7. | German; general review of antibacterial therapy for gram-positive bacterial infections; antibacterial activity and resistance; linezolid and telithromycin |
| Krige JE, Lindfield K, Friedrich L, Otradovec C, Martone WJ, Katz DE, et al. Effectiveness and duration of daptomycin therapy in resolving clinical symptoms in the treatment of complicated skin and skin structure infections. Curr Med Res Opin. 2007;23:2147-56. | Post hoc analysis of a subset of patients from two prospective trials |
| Laible BR. Vancomycin in the treatment of serious methicillin-resistant Staphylococcus aureus infections: time to move on to alternative agents? S D Med. 2011;64:465, 467. | General article on possible alternatives to vancomycin |
| Laohavaleeson S, Barriere SL, Nicolau DP, Kuti JL. Cost-effectiveness of telavancin versus vancomycin for treatment of complicated skin and skin structure infections. Pharmacotherapy. 2008;28:1471-82. | NHS EED abstract; cost-effectiveness analysis of treatments for MRSA-associated cSSSIs |
| Laohavaleeson S, Kuti JL, Nicolau DP. Telavancin: a novel lipoglycopeptide for serious gram-positive infections. Expert Opin Investig Drugs. 2007;16:347-57. | Not an SR (no methods reported) |
| LaPlante KL, Rybak MJ. Daptomycin - a novel antibiotic against Gram-positive pathogens. Expert Opin Pharmacother. 2004;5:2321-31. | Not an SR (no methods reported) |
| LaRosa SP, Tomecki KJ. Antibiotics: what's new? Cutis. 2004;73:10-4. | Not an SR (no methods reported) |
| Leach KL, Brickner SJ, Noe MC, Miller PF. Linezolid, the first oxazolidinone antibacterial agent. Ann N Y Acad Sci. 2011;1222:49-54. | General review of oxazolidinone agents, in particular linezolid |
| Lee SY, Kuti JL, Nicolau DP. Antimicrobial management of complicated skin and skin structure infections in the era of emerging resistance. Surg Infect (Larchmnt). 2005;6:283-95. | Not an SR (has search strategy but no other methods described) |
| Lenoble M. [Treatment of resistant Gram-positive bacterial infections]. Med Mal Infect. 2011;41 Suppl:1-6. | French; general review of treatment of gram-positive resistant bacterial infections |
| Leonard SN, Rybak MJ. Telavancin: an antimicrobial with a multifunctional mechanism of action for the treatment of serious gram-positive infections. Pharmacotherapy. 2008;28:458-68. | Not an SR (has search but no other methods reported) |
| Lerma FA. Linezolid in the treatment of gram positive coccal infections in critical patients. Rev Esp Quimioter. 2010;23:1-3. | Spanish; editorial discussing linezolid for treatment of gram-positive infections in critically ill patients |
| Levine JF. Vancomycin: a review. Med Clin North Am. 1987;71:1135-45. | Not an SR (no methods reported) |
| Li JZ, Willke RJ, Balan DA, Nathwani D, Lopez H, Rittenhouse BE, et al. Cost effectiveness analysis of linezolid vs. teicoplanin for the treatment of serious gram-positive bacterial infections in a multinational randomized trial. Value Health. 2003;6:259-60. | Conference abstract |
| Li JZ, Willke RJ, Rittenhouse BE, Glick HA. Approaches to analysis of length of hospital stay related to antibiotic therapy in a randomized clinical trial: linezolid versus vancomycin for treatment of known or suspected methicillin-resistant Staphylococcus species infections. Pharmacotherapy. 2002;22:45S-54S. | Not outcome of interest (length of stay) |
| Li JZ, Willke RJ, Rittenhouse BE, Rybak MJ. Effect of linezolid versus vancomycin on length of hospital stay in patients with complicated skin and soft tissue infections caused by known or suspected methicillin-resistant staphylococci: results from a randomized clinical trial. Surg Infect (Larchmnt). 2003;4:57-70. | Not valid outcomes (length of hospital stay); not primary publication |
| Li Z, Willke RJ, Pinto LA, Rittenhouse BE, Rybak MJ, Pleil AM, et al. Comparison of length of hospital stay for patients with known or suspected methicillin-resistant Staphylococcus species infections treated with linezolid or vancomycin: a randomized, multicenter trial. Pharmacotherapy. 2001;21:263-74. | No outcomes of interest (length of stay, weekly discharge and days of antibiotic treatment |
| Lim L, Sutton E, Brown J. Ceftaroline: a new broad-spectrum cephalosporin. Am J Health Syst Pharm. 2011;68:491-8. | Not an SR (no methods reported) |
| Linden P. Use of linezolid for gram-positive infections. Infect Med. 2002;19:25-32. | Narrative review of linezolid |
| Linezolid for gram-positive infections. Drug Ther Bull. 2001;39:54-6. | Not an SR (no methods reported) |
| Lipsky BA, Stoutenburgh U. Daptomycin for treating infected diabetic foot ulcers: evidence from a randomized, controlled trial comparing daptomycin with vancomycin or semi-synthetic penicillins for complicated skin and skin-structure infections. J Antimicrob Chemother. 2005;55:240-5. | Subset of diabetic patients from two RCTs of cSSTIs |
| Lipsky BA, Itani KMF, Weigelt JA, Joseph W, Paap CM, Reisman A, et al. The role of diabetes mellitus in the treatment of skin and skin structure infections caused by methicillin-resistant Staphylococcus aureus: results from three randomized controlled trials. Int J Infect Dis. 2011;15:e140-6. | Meta-analysis but no evidence of a systematic search for the included studies |
| Liu C, Bayer A, Cosgrove SE, Daum RS, Fridkin SK, Gorwitz RJ, et al. Clinical practice guidelines by the Infectious Diseases Society of America for the treatment of methicillin-resistant Staphylococcus aureus infections in adults and children: executive summary. Clin Infect Dis. 2011;52:285-92. | Not an RCT or SR |
| Logman JF, Stephens J, Heeg B, Haider S, Cappelleri J, Nathwani D, et al. Comparative effectiveness of antibiotics for the treatment of MRSA complicated skin and soft tissue infections. Curr Med Res Opin. 2010;(7):1565-78. | DARE abstract; full report already included |
| Lopez H, Li JZ, Balan DA, Willke RJ, Rittenhouse BE, Mozaffari E, et al. Hospital resource use and cost of treatment with linezolid versus teicoplanin for treatment of serious gram-positive bacterial infections among hospitalized patients from South America and Mexico: results from a multicenter trial. Clin Ther. 2003;25:1846-71. | Economic evaluation with some efficacy results |
| Luke D, Hewlett D Jr, Welch V, Chambers R, Huang D. Incidence of intravenous catheter-site complications in patients treated with linezolid or vancomycin for skin infections caused by methicillin-resistant Staphylococcus aureus. Hosp Pharm. 2011;46:427-31. | Post hoc analysis of AE frequency that excluded patients with baseline bacteremia and those who started on PO linezolid |
| Lyseng-Williamson KA, Blick SKA. Telavancin. Drugs. 2009;69:2607-20. | Drug profile of telavancin |
| Marchese A, Schito GC. The oxazolidinones as a new family of antimicrobial agent. Clin Microbiol Infect. 2001;7 Suppl 4:66-74. | General review of oxazolidinones: action, resistance, pharmacology, clinical and microbiological efficacy and safety |
| Marot JC, Jonckheere S, Munyentwali H, Belkhir L, Vandercam B, Yombi JC. Tigecycline-induced acute pancreatitis: about two cases and review of the literature. Acta Clin Belg. 2012;67:229-32. | Case report with discussion of tigecycline-induced pancreatitis |
| Mavros MN, Tansarli GS, Vardakas KZ, Rafailidis PI, Karageorgopoulos DE, Falagas ME. Impact of vancomycin minimum inhibitory concentration on clinical outcomes of patients with vancomycin-susceptible Staphylococcus aureus infections: a meta-analysis and meta-regression. Int J Antimicrob Agents. 2012;40:496-509. | SRSR: very broad, any study design |
| McAuley L. Linezolid for the treatment of serious gram-positive infections. Issues Emerg Health Technol. 2001; Mar:1-6. | Not an SR or RCT |
| McAuley L. Linezolid for the treatment of serious gram-positive infections. Health Technol Assess Database. 2001;(1):4. | Structured abstract |
| McCollum M, Sorensen SV, Liu LZ. A comparison of costs and hospital length of stay associated with intravenous/oral linezolid or intravenous vancomycin treatment of complicated skin and soft-tissue infections caused by suspected or confirmed methicillin-resistant Staphylococcus aureus in elderly US patients. Clin Ther. 2007;29:469-77. | Cost analysis for subset of elderly patients with MRSA cSSTI enrolled in linezolid cSSTI study |
| McGovern P, Babinchak T, Quintana A. Clarification to the Systematic Review and Meta-Analysis Involving Tigecycline. Antimicrob Agents Chemother. 2011;55:4941. | Correspondence relating to an SR/meta-analysis of tigecycline |
| McKinnon PS, Sorensen SV, Liu LZ, Itani KM. Impact of linezolid on economic outcomes and determinants of cost in a clinical trial evaluating patients with MRSA complicated skin and soft-tissue infections. Ann Pharmacother. 2006;40:1017-23. | Subset of US subjects enrolled in a multinational trial (pharmacoeconomic analysis but reports cure rate) |
| McKinnon PS, Sorensen SV, Liu LZ, Itani KMF. Impact of linezolid on economic outcomes and determinants of cost in a clinical trial evaluating patients with MRSA complicated skin and soft-tissue infections. Ann Pharmacother. 2006;40:1017-23. | Cost-effectiveness analysis of US patients enrolled in a multinational trial |
| Mendoza N, Tyring SK. Emerging drugs for complicated skin and skin-structure infections. Expert Opin Emerg Drugs. 2010;15:509-20. | Not an SR (no methodology) |
| Micek ST. Alternatives to vancomycin for the treatment of methicillin-resistant Staphylococcus aureus infections. Clin Infect Dis. 2007;45 Suppl 3:S184-90. | General review of pharmacology of drugs with activity against MRSA |
| Milatovic D. Vancomycin for treatment of infections with methicillin-resistant Staphylococcus aureus: are there alternatives? Eur J Clin Microbiol. 1986;5:689-92. | General review; not an SR |
| Mitrano JA, Spooner LM, Belliveau P. Excretion of antimicrobials used to treat methicillin-resistant Staphylococcus aureus infections during lactation: safety in breastfeeding infants. Pharmacotherapy. 2009;29:1103-9. | Focus on safety in breastfeeding infants |
| Moellering RC Jr. Current treatment options for community-acquired methicillin-resistant Staphylococcus aureus infection. Clin Infect Dis. 2008;46:1032-7. | General review of current treatments for community-acquired MRSA infections |
| Moise PA, North D, Steenbergen JN, Sakoulas G. Susceptibility relationship between vancomycin and daptomycin in Staphylococcus aureus: facts and assumptions. Lancet Infect Dis. 2009;9:617-24. | General review of microbiological effects of glycopeptides on bacterial infections and drug activity |
| Moran GJ, Abrahamian FM, Lovecchio F, Talan DA. Acute bacterial skin infections: developments since the 2005 Infectious Diseases Society of America (IDSA) guidelines. J Emerg Med. 2013;44:e397-412. | General review of developments in management/treatment of ABSSSI since 2005 guidelines |
| Nannini EC, Stryjewski ME. Pharmacotherapy update: daptomycin in the management of complicated skin and soft-tissue infections. Clin Med Insights Ther. 2010;2:453-60. | Non-SR of activity, resistance, pharmacokinetics and clinical use of daptomycin in SSSI |
| Nannini EC, Stryjewski ME, Corey GR. Ceftaroline for complicated skin and skin-structure infections. Expert Opin Pharmacother. 2010;11:1197-206. | General review of ceftaroline properties: in vitro activity, pharmacology, efficacy and safety observed in the clinical trials of patients with SSSI |
| Napolitano LM. Early appropriate parenteral antimicrobial treatment of complicated skin and soft tissue infections caused by methicillin-resistant Staphylococcus aureus. Surg Infect (Larchmnt). 2008;9 Suppl 1:s17-27. | General review of factors affecting choice of treatments for MRSA cSSTIs, available drugs and those under development |
| Napolitano LM. Emerging issues in the diagnosis and management of infections caused by multi-drug-resistant, gram-positive cocci. Surg Infect (Larchmnt). 2005;6 Suppl 2:S-5-22. | Not an SR (no methods reported) |
| Nathwani D, Li JZ, Balan DA, Willke RJ, Rittenhouse BE, Mozaffari E, et al. An economic evaluation of a European cohort from a multinational trial of linezolid versus teicoplanin in serious Gram-positive bacterial infections: the importance of treatment setting in evaluating treatment effects. Int J Antimicrob Agents. 2004;23:315-24. | Economic evaluation of factors affecting costs and length of stay in treatment of gram-positive bacterial infections |
| Nathwani D. Health economic issues in the treatment of drug-resistant serious Gram-positive infections. J Infect. 2009;59 Suppl 1:S40-50. | General review of economic impact of hospital-onset and hospital-acquired drug-resistant gram-positive infections |
| Nathwani D. Impact of methicillin-resistant Staphylococcus aureus infections on key health economic outcomes: does reducing the length of hospital stay matter? J Antimicrob Chemother. 2003;51 Suppl 2:ii37-44. | General review of the health economic impact of MRSA infections |
| Nathwani D. New antibiotics for the management of complicated skin and soft tissue infections: are they any better? Int J Antimicrob Agents. 2009;34 Suppl 1:S24-9. | Non-systematic comparative review of new parenteral agents with anti-MRSA activity vs more established drugs (e.g. vancomycin) |
| Nathwani D. Tigecycline: clinical evidence and formulary positioning. Int J Antimicrob Agents. 2005;25:185-92. | Review of clinical and microbiological efficacy and pharmacokinetic and safety data from key clinical trials of tigecycline in cSSTIs and IAIs |
| Neville LO, Brumfitt W, Hamilton-Miller JM, Harding I. Teicoplanin vs vancomycin for the treatment of serious infections: a randomised trial. Int J Antimicrob Agents. 1995;5:187-93. | Patients with gram-positive infections; not specifically MRSA-associated; two patients of relevance to current study? |
| Nicolau DP. Management of complicated infections in the era of antimicrobial resistance: the role of tigecycline. Expert Opin Pharmacother. 2009;10:1213-22. | Drug evaluation article summarizing the clinical utility of tigecycline, as demonstrated in clinical studies, reviews and case reports |
| Niu S-C, Deng S-T, Lee M-H, Ho C, Chang H-Y, Liu F-H. Modified vancomycin dosing protocol for treatment of diabetic foot infections. Am J Health Syst Pharm. 2008;65:1740-3. | Not an RCT. Prospective study of patients treated with either the conventional or modified dosing protocol |
| Norrby R. Linezolid--a review of the first oxazolidinone. Expert Opin Pharmacother. 2001;2:293-302. | General review; not an SR |
| Noskin GA. Tigecycline: a new glycylcycline for treatment of serious infections. Clin Infect Dis. 2005;41 Suppl 5:S303-14. | General review of microbiology and pharmacology of tigecycline for gram-positive, gram-negative and other infections |
| Oberholzer CM, Caserta MT. Antimicrobial update: daptomycin. Pediatr Infect Dis J. 2005;24:919-20. | Conference abstract |
| Padmanabhan RA, Larosa SP, Tomecki KJ. What's new in antibiotics? Dermatol Clin. 2005;23:301-12. | Looks like a narrative review (nothing suggesting SR in abstract) |
| Paladino JA. Linezolid: an oxazolidinone antimicrobial agent. Am J Health Syst Pharm. 2002;59:2413-25. | Not an SR (no methods reported) |
| Papanas N, Mani R. Advances in infections and wound healing for the diabetic foot: the die is cast. Int J Low Extrem Wounds. 2013;12:83-6. | General review of progress achieved in the treatment of diabetic foot infections and wound healing |
| Paradisi F, Corti G. [The role of linezolid in the therapy of infections caused by multiresistant gram-positive cocci]. Infez Med. 2004;12:19-26. | Italian; general review of activity, pharmacology, efficacy, safety and costs of linezolid for treatment of multi-resistant enterococcal and staphylococcal bacterial infections |
| Patanwala AE, Erstad BL, Nix DE. Cost-effectiveness of linezolid and vancomycin in the treatment of surgical site infections. Curr Med Res Opin. 2007;23:185-93. | Cost-effectiveness analysis |
| Peng Y, Ye X, Li Y, Bu T, Chen X, Bi J, et al. Teicoplanin as an effective alternative to vancomycin for treatment of MRSA infection in Chinese population: a meta-analysis of randomized controlled trials. PLoS One. 2013;8:e79782. | Meta-analysis of studies of patients with MRSA infection (no specific details); some included studies definitely pneumonia |
| Periti P, Stringa G, Donati L, Mazzei T, Mini E, Novelli A. Teicoplanin--its role as systemic therapy of burn infections and as prophylaxis for orthopaedic surgery. Italian Study Groups for Antimicrobial Prophylaxis in Orthopaedic Surgery and Burns. Eur J Surg Suppl. 1992;(567):3-8. | Neither study involves patients specifically with MRSA-associated infections |
| Peterson LR. A review of tigecycline--the first glycylcycline. Int J Antimicrob Agents. 2008;32 Suppl 4:S215-22. | Non-SR of pharmacology and clinical efficacy of tigecycline in cSSTIs and cIAIs |
| Pettigrew M, Thirion DJ, Libman M, Zanotti G. Cost comparison of linezolid versus vancomycin for treatment of complicated skin and skin-structure infection caused by methicillin-resistant Staphylococcus aureus in Quebec. Can J Infect Dis Med Microbiol. 2012;23:187-95. | Cost analysis for treatment of cSSSI due to MRSA |
| Phoenix G, Das S, Joshi M. Diagnosis and management of cellulitis. BMJ. 2012;345:e4955. | General review of cellulitis: causes, risk factors, prevention, diagnosis and management |
| Plosker GL, Figgitt DP. Linezolid: a pharmacoeconomic review of its use in serious Gram-positive infections. Pharmacoeconomics. 2005;23:945-64. | Not an SR (has search strategy but inclusion mainly based on methods section of the trials; no further details given) |
| Plouffe JF. Emerging therapies for serious gram-positive bacterial infections: a focus on linezolid. Clin Infect Dis. 2000;31 Suppl 4:S144-9. | General review on antimicrobial resistance and emerging therapies, in particular clinical use of linezolid |
| Poon H, Chang MH, Fung HB. Ceftaroline fosamil: a cephalosporin with activity against methicillin-resistant Staphylococcus aureus. Clin Ther. 2012;34:743-65. | Comparator is a combination |
| Poulakou G, Giamarellou H. Investigational treatments for postoperative surgical site infections. Expert Opin Investig Drugs. 2007;16:137-55. | Not an SR (no methods reported) |
| Prokocimer P, Bien P, Deanda C, Pillar CM, Bartizal K. In vitro activity and microbiological efficacy of tedizolid (TR-700) against Gram-positive clinical isolates from a phase 2 study of oral tedizolid phosphate (TR-701) in patients with complicated skin and skin structure infections. Antimicrob Agents Chemother. 2012;56:4608-13. | Not an RCT or SR. Report of a dosing study |
| Quist SR, Fierlbeck G, Seaton RA, Loeffler J, Chaves RL. Comparative randomised clinical trial against glycopeptides supports the use of daptomycin as first-line treatment of complicated skin and soft-tissue infections. Int J Antimicrob Agents. 2012;39:90-1. | Letter reporting randomized trial of daptomycin in cSSTIs |
| Rafailidis PI, Kouranos VD, Christodoulou C, Falagas ME. Linezolid for patients with neutropenia: are bacteriostatic agents appropriate? Expert Rev Anti Infect Ther. 2009;7:415-22. | SR of studies of bacterial infections in patients with neutropenia |
| Raghavan M, Linden PK. Newer treatment options for skin and soft tissue infections. Drugs. 2004;64:1621-42. | Not an SR (no methods reported) |
| Rahman M. Alternatives to vancomycin in treating methicillin-resistant Staphylococcus aureus infections. J Antimicrob Chemother. 1998;41:325-8. | Not an SR |
| Ramsey TD, Lau TT, Ensom MH. Serotonergic and adrenergic drug interactions associated with linezolid: a critical review and practical management approach. Ann Pharmacother. 2013;47:543-60. | SRSR of drug interactions in studies co-administering serotonergic or adrenergic agents with linezolid |
| Rose WE, Rybak MJ. Tigecycline: first of a new class of antimicrobial agents. Pharmacotherapy. 2006;26:1099-110. | Not an SR (no methods reported) |
| Rosner AJ, Becker DL, Wong AH, Miller E, Conly JM. The costs and consequences of methicillin-resistant Staphylococcus aureus infection treatments in Canada. Can J Infect Dis Med Microbiol. 2004;15:213-20. | Not an RCT: chart review and cost-consequences analysis of patient treated for active MRSA SSTI |
| Rubinstein E, Corey GR, Stryjewski ME, Kanafani ZA. Telavancin for the treatment of serious gram-positive infections, including hospital acquired pneumonia. Expert Opin Pharmacother. 2011;12:2737-50. | Not an SR (no review methods reported) |
| Rubinstein E, Isturiz R, Standiford HC, Smith LG, Oliphant TH, Cammarata S, et al. Worldwide assessment of linezolid's clinical safety and tolerability: comparator-controlled phase III studies. Antimicrob Agents Chemother. 2003;47:1824-31. | Pooled safety data from phase 3 clinical trials; no evidence of a systematic search for included trials |
| Rybak MJ. The efficacy and safety of daptomycin: first in a new class of antibiotics for Gram-positive bacteria. Clin Microbiol Infect. 2006;12 Suppl 1:24-32. | General review of microbiology, pharmacology, efficacy and safety of daptomycin in gram-positive bacterial infections, including cSSSI |
| Sabol KE, Echevarria KL, Lewis JS 2nd. Community-associated methicillin-resistant Staphylococcus aureus: new bug, old drugs. Ann Pharmacother. 2006;40:1125-33. | Community-acquired MRSA infections, not specifically cSSSI |
| Santayana EM, Jourjy J. Treatment of methicillin-resistant Staphylococcus aureus surgical site infections. AACN Adv Crit Care. 2011;22:5-12; quiz 14. | General review; not an SR |
| Saravolatz LD, Stein GE, Johnson LB. Ceftaroline: a novel cephalosporin with activity against methicillin-resistant Staphylococcus aureus. Clin Infect Dis. 2011;52:1156-63. | Non-SR of properties, action, safety and efficacy of ceftaroline |
| Sauermann R, Rothenburger M, Graninger W, Joukhadar C. Daptomycin: a review 4 years after first approval. Pharmacology. 2008;81:79-91. | Review of history, mechanism of action, susceptibility, recent discoveries and clinical experience and current role of daptomycin in infectious diseases |
| Schafer JJ, Goff DA. Establishing the role of tigecycline in an era of antimicrobial resistance. Expert Rev Anti Infect Ther. 2008;6:557-67. | Drug profile on tigecycline: chemistry, microbiology, pharmacology, resistance, efficacy and safety. Also commentary and 5-year view |
| Scheinfeld N. A comparison of available and investigational antibiotics for complicated skin infections and treatment-resistant Staphylococcus aureus and enterococcus. J Drugs Dermatol. 2007;6:97-103. | Not an SR |
| Scheinfeld N. Infections in the elderly. Dermatol Online J. 2005;11:8. | General review of skin infections occurring in the elderly |
| Schmidt-Ioanas M, de Roux A, Lode H. New antibiotics for the treatment of severe staphylococcal infection in the critically ill patient. Curr Opin Crit Care. 2005;11:481-6. | Not an SR (no methods reported) |
| Schurmann D, Sorensen SV, De Cock E, Duttagupta S, Resch A. Cost-effectiveness of linezolid versus vancomycin for hospitalised patients with complicated skin and soft-tissue infections in Germany. Eur J Health Econ. 2009;10:65-79. | Cost-effectiveness analysis of treatments for MRSA-associated cSSTI |
| Schweiger ES, Weinberg JM. Novel antibacterial agents for skin and skin structure infections. J Am Acad Dermatol. 2004;50:331-40; quiz 341-2. | Continuing medical education article on antibacterial agents for SSSIs: action, pharmacology and efficacy, safety and dosing |
| Schweiger ES, Scheinfeld NS, Tischler HR, Weinberg JM. Linezolid and quinupristin/dalfopristin: novel antibiotics for gram-positive infections of the skin. J Drugs Dermatol. 2003;2:378-83. | Not an SR (no methods reported) |
| Seputiene V, Povilonis J, Armalyte J, Suziedelis K, Pavilonis A, Suziedeliene E. Tigecycline - how powerful is it in the fight against antibiotic-resistant bacteria? Medicina (Kaunas). 2010;46:240-8. | General review of molecular mechanism of action, activity, antibiotic resistance and clinical application of tigecycline |
| Shah PM. The need for new therapeutic agents: what is the pipeline? Clin Microbiol Infect. 2005;11 Suppl 3:36-42. | Not an SR (no methods reported) |
| Shaw KJ, Barbachyn MR. The oxazolidinones: past, present, and future. Ann N Y Acad Sci. 2011;1241:48-70. | General review of the oxazolidinone class of drugs |
| Shea KW, Cunha BA. Teicoplanin. Med Clin North Am. 1995;79:833-44. | Not an SR (no methods reported) |
| Shorr AF. Epidemiology and economic impact of meticillin-resistant Staphylococcus aureus: review and analysis of the literature. Pharmacoeconomics. 2007;25:751-68. | Review of economic evaluations |
| Slover CM, Rodvold KA, Danziger LH. Tigecycline: a novel broad-spectrum antimicrobial. Ann Pharmacother. 2007;41:965-72. | Non-SR of efficacy and safety of tigecycline in cSSSI and cIAI |
| Smith WJ, Drew RH. Telavancin: a new lipoglycopeptide for gram-positive infections. Drugs Today. 2009;45:159-73. | General review summarizing data on the pharmacologic properties, antibacterial activity, clinical efficacy and safety of telavancin |
| Sorensen SV, Hollenbeak CS, Baker TM, Resch A, Duttagupta S. Linezolid for the treatment of skin and soft-tissue MRSA infections - a cost-effective alternative to vancomycin: evidence from a multinational clinical trial. Value Health. 2004;7:758-9. | Conference abstract |
| Squires RA, Postier RG. Tigecycline for the treatment of infections due to resistant Gram-positive organisms. Expert Opin Investig Drugs. 2006;15:155-62. | Not an SR (no methods reported) |
| Stahl JP. [Epidemiology, control and treatments of antimicrobial resistances: highlights of the 45th ICAAC, Washington, 2005]. Med Mal Infect. 2006;36:290-6. | French; overview of presentations on epidemiology and management of hospital and community-acquired antimicrobial resistances |
| Steenbergen JN, Alder J, Thorne GM, Tally FP. Daptomycin: a lipopeptide antibiotic for the treatment of serious Gram-positive infections. J Antimicrob Chemother. 2005;55:283-8. | General review of microbiology, pharmacology and efficacy of daptomycin for gram-positive infections |
| Stein GE, Craig WA. Tigecycline: a critical analysis. Clin Infect Dis. 2006;43:518-24. | General review of microbiology, pharmacology, clinical efficacy, dosing and safety of tigecycline |
| Stein GE, Wells EM. The importance of tissue penetration in achieving successful antimicrobial treatment of nosocomial pneumonia and complicated skin and soft-tissue infections caused by methicillin-resistant Staphylococcus aureus: vancomycin and linezolid. Curr Med Res Opin. 2010;26:571-88. | Not an SR (has search and vague review question, but no other methods reported) |
| Stein GE, Schooley SL, Havlichek DH, Nix DE. Outpatient intravenous antibiotic therapy compared with oral linezolid in patients with skin and soft tissue infections: a pharmacoeconomic analysis. Infect Dis Clin Pract. 2008;16:235-9. | Cost-effectiveness analysis of OPAT vs linezolid |
| Stein RA. A new antibiotic: lessons and perspectives. Int J Clin Pract. 2008;62:1836-7. | Perspective on the problem of antibiotic resistance, how to address it and the role of emerging therapeutics |
| Strahilevitz J, Rubinstein E. Novel agents for resistant Gram-positive infections--a review. Int J Infect Dis. 2002;6 Suppl 1:S38-46. | General review of drugs for resistant gram-positive infections, focusing on linezolid, daptomycin, GAR-936 and oritavancin |
| Strain J. Daptomycin: overview of an innovative antibiotic. S D J Med. 2004;57:445-6. | Not an SR (no methods reported) |
| Stryjewski ME, Chambers HF. Skin and soft-tissue infections caused by community-acquired methicillin-resistant Staphylococcus aureus. Clin Infect Dis. 2008;46 Suppl 5:S368-77. | General review of community-acquired MRSA SSTIs and their treatment |
| Stryjewski ME, Corey GR. New treatments for methicillin-resistant Staphylococcus aureus. Curr Opin Crit Care. 2009;15:403-12. | Not an SR (no methods reported) |
| Stryjewski ME, Barriere SL, O'Riordan W, Dunbar LM, Hopkins A, Genter FC, et al. Efficacy of telavancin in patients with specific types of complicated skin and skin structure infections. J Antimicrob Chemother. 2012;67:1496-502. | Post hoc analysis of ATLAS trials; primary publication included |
| Stryjewski ME, Chu VH, O'Riordan WD, Warren BL, Dunbar LM, Young DM, et al. Telavancin versus standard therapy for treatment of complicated skin and skin structure infections caused by gram-positive bacteria: FAST 2 study. Antimicrob Agents Chemother. 2006;50:862-7. | Comparator is vancomycin or anti-staphylococcal penicillin; results not reported separately for those receiving telavancin |
| Stryjewski ME, O'Riordan WD, Lau WK, Pien FD, Dunbar LM, Vallee M, et al. Telavancin versus standard therapy for treatment of complicated skin and soft-tissue infections due to gram-positive bacteria. Clin Infect Dis. 2005;40:1601-7. | Participants with cSSTI caused by gram-positive organisms; results not broken down according to disease and pathogen |
| Svetitsky S, Leibovici L, Paul M. Comparative efficacy and safety of vancomycin versus teicoplanin: systematic review and meta-analysis. Antimicrob Agents Chemother. 2009;53:4069-79. | SR/meta-analysis of studies of vancomycin vs teicoplanin in gram-positive resistant bacterial infections; mixed populations (age, indication and bacterial species) |
| Talbot GH, O'Neal T, Das AF, Thye D. Prospective study of the Wilson severity-of-illness scoring system for complicated skin and skin structure infections. Antimicrob Agents Chemother. 2013;57:647-50. | Not original trial reports: validation of a severity-of-illness scoring system for cSSSI |
| Tally FP, DeBruin MF. Development of daptomycin for gram-positive infections. J Antimicrob Chemother. 2000;46:523-6. | Overview of drug development for daptomycin, including pharmacokinetics, animal studies and early trials of humans |
| Tasina E, Haidich AB, Kokkali S, Arvanitidou M. Efficacy and safety of tigecycline for the treatment of infectious diseases: a meta-analysis. Lancet Infect Dis. 2011;11:834-44. | No studies included of comparators of interest |
| Tedesco KL, Rybak MJ. Daptomycin. Pharmacotherapy. 2004;24:41-57. | Not an SR (no methods reported) |
| Telavancin (Vibativ) for gram-positive skin infections. Med Lett Drugs Ther. 2010;52:1-2. | Conference abstract |
| Telavancin: TD 6424, TD-6424. Drugs R D. 2006;7:384-8. | Not an SR |
| Thompson S, Townsend R. Pharmacological agents for soft tissue and bone infected with MRSA: which agent and for how long? Injury. 2011;42 Suppl 5:S7-10. | General review of pharmacological agents used to treat MRSA soft-tissue and bone infections |
| Townsend ML, Pound MW, Drew RH. Tigecycline in the treatment of complicated intra-abdominal and complicated skin and skin structure infections. Ther Clin Risk Manag. 2007;3:1059-70. | Non-SR of tigecycline in treatment of cSSSIs and complicated IAIs |
| Townsend ML, Pound MW, Drew RH. Tigecycline: a new glycylcycline antimicrobial. Int J Clin Pract. 2006;60:1662-72. | General review of pharmacology and clinical applications of tigecycline |
| Treatment of methicillin-resistant Staphylococcus aureus infections. Med Lett Drugs Ther. 1982;24:107-8. | Brief information on methicillin resistance, alternative antibiotics, clinical experience and precautions with vancomycin |
| Turina M, Cheadle WG. Clinical challenges and unmet needs in the management of complicated skin and skin structure, and soft tissue infections. Surg Infect (Larchmnt). 2005;6 Suppl 2:S-23-36. | Not an SR |
| Tverdek FP, Crank CW, Segreti J. Antibiotic therapy of methicillin-resistant Staphylococcus aureus in critical care. Crit Care Clin. 2008;24:249-60, vii-viii. | General review article of selected antibiotics for treating MRSA infections in critical care |
| Van Bambeke F, Mingeot-Leclercq M-P, Struelens MJ, Tulkens PM. The bacterial envelope as a target for novel anti-MRSA antibiotics. Trends Pharmacol Sci. 2008;29:124-34. | General review of mode of action, pharmacology, microbiology and target indications of anti-MRSA agents |
| Van der Auwera P, Aoun M, Meunier F. Randomized study of vancomycin versus teicoplanin for the treatment of gram-positive bacterial infections in immunocompromised hosts. Antimicrob Agents Chemother. 1991;35:451-7. | Patients with gram-positive infections, including SSTI, caused by various pathogens, but not specifically MRSA |
| van Hal SJ, Paterson DL. New Gram-positive antibiotics: better than vancomycin? Curr Opin Infect Dis. 2011;24:515-20. | General review focusing on alternative antibiotics to vancomycin for the treatment of multidrug-resistant gram-positive infections |
| Van Laethem Y, Hermans P, De Wit S, Goosens H, Clumeck N. Teicoplanin compared with vancomycin in methicillin-resistant Staphylococcus aureus infections: preliminary results. J Antimicrob Chemother. 1988;21 Suppl A:81-7. | Only three patients with condition relevant to current review (post-operative cellulitis; see Table II)? |
| Vanderauwera P, Aoun M, Meunier F. Randomized study of vancomycin versus teicoplanin for the treatment of Gram-positive bacterial-infections in immunocompromised hosts. Antimicrob Agents Chemother. 1991;35:451-7. | Patients with gram-positive infections, including bacteremia and SSTIs, not specifically MRSA |
| Vardakas KZ, Ntziora F, Falagas ME. Linezolid: effectiveness and safety for approved and off-label indications. Expert Opin Pharmacother. 2007;8:2381-400. | Not an SR (no methods reported) |
| Vilhena C, Bettencourt A. Daptomycin: a review of properties, clinical use, drug delivery and resistance. Mini Rev Med Chem. 2012;12:202-9. | General review of evidence on daptomycin molecular structure, mechanism of action, bacterial spectrum, clinical uses, local delivery, toxicity and resistance |
| Vinh DC, Rubinstein E. Linezolid: a review of safety and tolerability. J Infect. 2009;59 Suppl 1:S59-74. | Non-SR of safety and tolerability of linezolid |
| Vinken AG, Li JZ, Balan DA, Rittenhouse BE, Willke RJ, Goodman C. Comparison of linezolid with oxacillin or vancomycin in the empiric treatment of cellulitis in US hospitals. Am J Ther. 2003;10:264-74. | NHS EED abstract reporting cost-effectiveness analysis |
| Weber DJ, Rutala WA. New antibiotic agents: problems and prospects. Surg Infect (Larchmnt). 2005;6 Suppl 2:S-97-107. | Not an SR |
| Wesson KM, Lerner DS, Silverberg NB, Weinberg JM. Linezolid, quinupristin/dalfopristin, and daptomycin in dermatology. Dis Mon. 2004;50:395-406. | General review of mode of action, pharmacology, efficacy and dosing of selected treatments for resistant gram-positive organisms |
| Wesson KM, Lerner DS, Silverberg NB, Weinberg JM. Linezolid, quinupristin/dalfopristin, and daptomycin in dermatology. Clin Dermatol. 2003;21:64-70. | General review of pharmacology, efficacy, safety and dosing of selected treatments for gram-positive infections |
| Wilcox MH, Tack KJ, Bouza E, Herr DL, Ruf BR, Ijzerman MM, et al. Complicated skin and skin-structure infections and catheter-related bloodstream infections: noninferiority of linezolid in a phase 3 study. Clin Infect Dis. 2009;48:203-12. | Potential difference noted in the initial course of therapy as a result of the suspicion of catheter-related bloodstream infection at study onset |
| Wilcox MH. Efficacy of linezolid versus comparator therapies in Gram-positive infections. J Antimicrob Chemother. 2003;51 Suppl 2:ii27-35. | Non-SR summarizing phase 3 study evidence of linezolid vs comparators for gram-positive infections, including SSTIs and MRSA infections |
| Wilcox MH. Efficacy of tigecycline in complicated skin and skin structure infections and complicated intra-abdominal infections. J Chemother. 2005;17 Suppl 1:23-9. | Length of stay, readmission rates and treatment duration not valid outcomes and not reported separately according to type of cSSTI |
| Wilcox MH. MRSA new treatments on the horizon: current status. Injury. 2011;42 Suppl 5:S42-4. | General review of treatments for MRSA infections such as cSSSI |
| Wilcox MH. Update on linezolid: the first oxazolidinone antibiotic. Expert Opin Pharmacother. 2005;6:2315-26. | Not an SR (no methods reported) |
| Willke RJ, Glick HA, Li JZ, Rittenhouse BE. Effects of linezolid on hospital length of stay compared with vancomycin in treatment of methicillin-resistant Staphylococcus infections. An application of multivariate survival analysis. Int J Technol Assess Health Care. 2002;18:540-54. | Length of stay is not an outcome of interest |
| Wilson AP, Gruneberg RN, Neu H. A critical review of the dosage of teicoplanin in Europe and the United States. Int J Antimicrob Agents. 1994;4 Suppl 1:1-30. | Non-SR article (no methods) |
| Wilson P. Linezolid: a new antibiotic for gram-positive infections. Hosp Med. 2001;62:682-6. | Not an SR (no methodology) |
| Wilson SE, O'Riordan W, Hopkins A, Friedland HD, Barriere SL, Kitt MM, et al. Telavancin versus vancomycin for the treatment of complicated skin and skin-structure infections associated with surgical procedures. Am J Surg. 2009;197:791-6. | Retrospective pooled analysis of the two ATLAS trials |
| Wilson SE, Solomkin JS, Le V, Cammarata SK, Bruss JB. A severity score for complicated skin and soft tissue infections derived from phase III studies of linezolid. Am J Surg. 2003;185:369-75. | Not primary RCT (analyses based on two trials) |
| Wilson SE. Clinical trial results with linezolid, an oxazolidinone, in the treatment of soft tissue and postoperative gram-positive infections. Surg Infect (Larchmnt). 2001;2:25-35. | Not an SR (no methods reported) |
| Wong-Beringer A, Joo J, Tse E, Beringer P. Vancomycin-associated nephrotoxicity: a critical appraisal of risk with high-dose therapy. Int J Antimicrob Agents. 2011;37:95-101. | Semi-SR: has search and inclusion criteria, but no details of study selection/extraction process |
| Wood MJ. The comparative efficacy and safety of teicoplanin and vancomycin. J Antimicrob Chemother. 1996;37:209-22. | Meta-analysis of RCTs comparing vancomycin and teicoplanin in various indications; no evidence of a systematic search for included studies |
| Wu G, Abraham T, Rapp J, Vastey F, Saad N, Balmir E. Daptomycin: evaluation of a high-dose treatment strategy. Int J Antimicrob Agents. 2011;38:192-6. | Not an SR (extensive search but no other methods reported) |
| Yahav D, Lador A, Paul M, Leibovici L. Efficacy and safety of tigecycline: a systematic review and meta-analysis. J Antimicrob Chemother. 2011;66:1963-71. | SR/meta-analysis included adults or children with any infection; not specifically MRSA-associated or ABSSSI/cSSSI |
| Yang A, Kerdel FA. Infectious disease update: new anti-microbials. Semin Cutan Med Surg. 2006;25:94-9. | Not an SR (no methodology) |
| Zeckel ML. A closer look at vancomycin, teicoplanin, and antimicrobial resistance. J Chemother. 1997;9:311-31; discussion 332-5. | Not an SR (no methods reported) |
| Zhanel GG, Calic D, Schweizer F, Zelenitsky S, Adam H, Lagace-Wiens PR, et al. New lipoglycopeptides: a comparative review of dalbavancin, oritavancin and telavancin. Drugs. 2010;70:859-86. | Review of the chemistry, action, resistance, microbiology, pharmacology, efficacy and adverse effects of three antibiotics |
| Zhanel GG, Karlowsky JA, Rubinstein E, Hoban DJ. Tigecycline: a novel glycylcycline antibiotic. Expert Rev Anti Infect Ther. 2006;4:9-25. | General review; not an SR |
| Zhanel GG, Sniezek G, Schweizer F, Zelenitsky S, Lagace-Wiens PR, Rubinstein E, et al. Ceftaroline: a novel broad-spectrum cephalosporin with activity against meticillin-resistant Staphylococcus aureus. Drugs. 2009;69:809-31. | General review of ceftaroline: chemistry, action, resistance, microbiology, pharmacology, animal studies, clinical trials and adverse effects |
| Zhanel GG, Trapp S, Gin AS, DeCorby M, Lagace-Wiens PR, Rubinstein E, et al. Dalbavancin and telavancin: novel lipoglycopeptides for the treatment of Gram-positive infections. Expert Rev Anti Infect Ther. 2008;6:67-81. | General review; not an SR |
| Ziglam H, Nathwani D. New therapeutic agents for resistant Gram-positive infections. Expert Rev Anti Infect Ther. 2003;1:655-65. | Not an SR (no methods reported) |
| Ziglam H. Daptomycin and tigecycline: a review of clinical efficacy in the antimicrobial era. Expert Opin Pharmacother. 2007;8:2279-92. | Not an SR (no methods reported) |
| Ziglam HM, Finch RG. Limitations of presently available glycopeptides in the treatment of Gram-positive infection. Clin Microbiol Infect. 2001;7 Suppl 4:53-65. | General review focusing on the use and limitations (e.g. antibiotic resistance) of vancomycin and teicoplanin in clinical practice |

*ABSSSI* acute bacterial skin and skin structure infection, *AE* adverse event, *CABP* community-acquired bacterial pneumonia, *cIAI* complicated intra-abdominal infection, *cSSSI* complicated skin and skin structure infection, *cSSTI* complicated skin and soft tissue infection, *DARE* Database of Abstracts of Reviews of Effects, *EED* economic evaluation database, *FDA* US Food and Drug Administration, *IAI* intra-abdominal infection, *MRSA* methicillin-resistant *Staphylococcus aureus,* *NHS* UK National Health Service, *OPAT* outpatient parenteral antimicrobial therapy, *PO* per os (oral), *RCT* randomized controlled trial, *SAB* *Staphylococcus aureus* bacteremia, *SR* systematic review; *SRSR* survey research shared resource

# APPENDIX D WinBUGS code

**D.1 Network meta-analysis models**

The WinBUGS code for the fixed effect and random effects models are shown in this appendix.

***D.1.1 Fixed effect model***

# Fixed effect model

model{

# Model for log odds of outcome on each treatment arm

for(i in 1:N) {

logit(p[i])<-mu[s[i]]+ d[t[i]] - d[b[i]]

r[i]~dbin(p[i],n[i]) # Binomial distribution for outcome

}

# Vague priors for trial baselines

for(j in 1:NS) {mu[j]~dnorm(0,.0001)}

d[1]<-0

# Vague priors for basic parameters

for (k in 2:NT) {d[k] ~ dnorm(0,.0001)}

# Calculate odds ratios

# pairwise ORs

for (c in 1:(NT-1)){

for (k in (c+1):NT){

lor[c,k] <- d[k] - d[c]

log(or[c,k]) <- lor[c,k]

lor[k,c] <- d[c] - d[k]

log(or[k,c]) <- lor[k,c]

}

}

***D.1.2 Random Effects Model***

# Random effects model

model{

# Model for log odds of outcome on each treatment arm

for(i in 1:N) {

r[i]~dbin(p[i],n[i]) # Binomial distribution for outcome

logit(p[i])<-mu[s[i]]+ delta[i]

dd[i] <- d[t[i]]-d[b[i]]

delta[i] ~ dnorm(dd[i],bs.tau)

}

# Vague priors for trial baselines

for(j in 1:NS) {mu[j]~dnorm(0,.0001)}

d[1]<-0

# Vague priors for basic parameters

for (k in 2:NT) {d[k] ~ dnorm(0,.0001)}# used to be 0.0001

# Prior distribution for between study variance. Turner prior

tau ~ dlnorm(-2.34, 0.381039)

# prior for Subjective type (Turner et al 2012). Pharma v Pharma

bs.tau<-1/tau

# Calculate odds ratios

# pairwise ORs

for (c in 1:(NT-1)){

for (k in (c+1):NT){

lor[c,k] <- d[k] - d[c]

log(or[c,k]) <- lor[c,k]

lor[k,c] <- d[c] - d[k]

log(or[k,c]) <- lor[k,c]

}

}

**D.2 Input data for the models**

***D.2.1 Clinical response at the end of treatment (all trials)***

| **Study Reference** | **Treatment** | **Number of patients** | **Number of events** |
| --- | --- | --- | --- |
| Prokocimer 2013* | Tedizolid | 332 | 230 |
| Prokocimer 2013* | Linezolid | 335 | 241 |
| Itani 2010* | Linezolid | 284 | 254 |
| Itani 2010* | Vancomycin | 287 | 243 |
| Lin 2008 | Linezolid | 33 | 31 |
| Lin 2008 | Vancomycin | 26 | 19 |
| Kohno 2007 | Linezolid | 18 | 14 |
| Kohno 2007 | Vancomycin | 10 | 6 |
| Talbot 2007 | Ceftaroline | 61 | 60 |
| Talbot 2007 | Vancomycin | 27 | 26 |
| Wilcox 2004* | Linezolid | 117 | 113 |
| Wilcox 2004* | Teicoplanin | 111 | 103 |
| Moran 2014* | Tedizolid | 332 | 304 |
| Moran 2014* | Linezolid | 334 | 301 |

* Trials included in the ITT/mITT network

***D.2.2 Clinical response at the post treatment evaluation or test of cure (all trials)***

| **Study Reference** | **Treatment** | **Number of patients** | **Number of events** |
| --- | --- | --- | --- |
| Aikawa 2013* | Daptomycin | 55 | 45 |
| Aikawa 2013* | Vancomycin | 19 | 16 |
| Prokocimer 2013* | Tedizolid | 332 | 284 |
| Prokocimer 2013* | Linezolid | 335 | 288 |
| Itani 2010* | Linezolid | 276 | 223 |
| Itani 2010* | Vancomycin | 266 | 196 |
| Pertel 2009* | Daptomycin | 50 | 47 |
| Pertel 2009* | Vancomycin | 51 | 46 |
| Lin 2008 | Linezolid | 33 | 30 |
| Lin 2008 | Vancomycin | 24 | 19 |
| Florescu 2008* | Tigecycline | 70 | 55 |
| Florescu 2008* | Vancomycin | 23 | 20 |
| Stryjewski 2008* | Telavancin | 928 | 710 |
| Stryjewski 2008* | Vancomycin | 939 | 697 |
| Kohno 2007 | Linezolid | 17 | 9 |
| Kohno 2007 | Vancomycin | 10 | 5 |
| Talbot 2007* | Ceftaroline | 67 | 59 |
| Talbot 2007* | Vancomycin | 32 | 26 |
| Weigelt 2005* | Linezolid | 338 | 314 |
| Weigelt 2005* | Vancomycin | 326 | 287 |
| Sharpe 2005* | Linezolid | 30 | 29 |
| Sharpe 2005* | Vancomycin | 30 | 13 |
| Stevens 2002* | Linezolid | 122 | 64 |
| Stevens 2002* | Vancomycin | 108 | 54 |
| Evers 2013* | Daptomycin | 20 | 17 |
| Evers 2013* | Telavancin | 20 | 17 |
| Moran 2014* | Tedizolid | 332 | 292 |
| Moran 2014* | Linezolid | 334 | 293 |

* Trials included in the ITT/mITT network

***D.2.3 Clinical response at the post treatment evaluation or test of cure (MRSA only)***

| **Study Reference** | **Treatment** | **Number of patients** | **Number of events** |
| --- | --- | --- | --- |
| Aikawa 2013 | Daptomycin | 55 | 45 |
| Aikawa 2013 | Vancomycin | 19 | 16 |
| Prokocimer 2013 | Tedizolid | 88 | 75 |
| Prokocimer 2013 | Linezolid | 90 | 77 |
| Itani 2010 | Linezolid | 276 | 223 |
| Itani 2010 | Vancomycin | 266 | 196 |
| Florescu 2008 | Tigecycline | 70 | 55 |
| Florescu 2008 | Vancomycin | 23 | 20 |
| Stryjewski 2008 | Telavancin | 278 | 252 |
| Stryjewski 2008 | Vancomycin | 301 | 260 |
| Kohno 2007 | Linezolid | 17 | 9 |
| Kohno 2007 | Vancomycin | 10 | 5 |
| Stevens 2002 | Linezolid | 60 | 37 |
| Stevens 2002 | Vancomycin | 51 | 32 |
| **Additional data for post hoc sensitivity analysis** | | | |
| Talbot 2007 | Ceftaroline | 5 | 4 |
| Talbot 2007 | Vancomycin | 5 | 5 |
| Corey 2010 | Ceftaroline | 179 | 155 |
| Corey 2010 | Vancomycin (plus aztreonam) | 151 | 124 |

***D.2.4 Discontinuation due to adverse events (all trials)***

| **Study Reference** | **Treatment** | **Number of patients** | **Number of events** |
| --- | --- | --- | --- |
| Aikawa 2013 | Vancomycin | 22 | 0 |
| Aikawa 2013 | Daptomycin | 88 | 1 |
| Prokocimer 2013 | Linezolid | 335 | 2 |
| Prokocimer 2013 | Tedizolid | 331 | 2 |
| Pertel 2009 | Vancomycin | 51 | 1 |
| Pertel 2009 | Daptomycin | 50 | 0 |
| Stryjewski 2008 | Vancomycin | 938 | 53 |
| Stryjewski 2008 | Telavancin | 929 | 73 |
| Talbot 2007 | Vancomycin | 32 | 1 |
| Talbot 2007 | Ceftaroline | 67 | 2 |
| Weigelt 2005 | Vancomycin | 588 | 34 |
| Weigelt 2005 | Linezolid | 592 | 30 |
| Moran 2014 | Linezolid | 334 | 4 |
| Moran 2014 | Tedizolid | 332 | 1 |

**APPENDIX E Similarity assessment**

**E.1 Assessment of similarity of trials for indirect comparison**

**Table E.1** Quality assessment

*Data presented in italics within this table refer to a population including patients with infection types other than skin structure infections.*

| Study reference | Randomization | Blinding | ITT analysis and methods used to impute missing data | Study duration and follow-up  a: Pre-treatment,  b: Treatment phase,  c: Post follow-up | Loss to follow-up |
| --- | --- | --- | --- | --- | --- |
| Aikawa 2013 [22] | Patients were randomly assigned regardless of the causative pathogen, but method of randomization was not described | No blinding of patients or treatment providers (open label). Outcome assessors were blinded to the treatment intervention | ITT analysis was conducted on 110/111 randomly assigned patients (safety population). The primary efficacy analysis was conducted in the mITT-MRSA population (74 patients), all patients who received at least one dose of study drug and in whom MRSA infection was confirmed on screening cultures. No missing data imputation methods were reported | a: NR  b:7–14 days c: Clinical responses at TOC within 7–14 days of the last treatment dose | None reported. 1/111 patients was randomly assigned but not treated, no explanation given |
| Evers 2013 [29] | NR | Single blinded | NR. No mention of missing data | a: NR  b: 10–14 days (telavancin) or "for an average of 10–14 days" (daptomycin) c: 2–4 weeks after cessation of treatment | NR |
| Florescu 2008 [32] | The randomization schedule was generated by the Biostatistics Section of Wyeth Research and accessed through a centralized computerized randomization system. Subjects were stratified at the time of randomization by type of infection, site of infection and APACHE II score (>15 or ≤15) | Double blind. After a subject was screened and deemed eligible for the study, the unblinded dispenser called the provided telephone number to determine the treatment assignment | ITT analysis was used for safety analysis (AEs). For clinical and microbiological response, an m-mITT population was analyzed | a: One screening visit 24 hours before the start of therapy b: Up to 28 days of study drug administration c: One post-therapy visit between 12 and 37 days after the last dose of the study drug for the TOC assessment | NR |
| Itani 2010 [23] | NR | Open label | ITT analysis was used for AE rate but not for the assessment of response (an mITT for MRSA confirmed cases was used). Missing data were not imputed | a: NR  b: 7–14 days c: Up to 7–10 days after the last dose of study medication | "Missing/indeterminate" clinical outcome:  EOT (within 72 hours after discontinuation of medication)  Linezolid: 2/240 (0.8 %);  Vancomycin: 3/222 (1.4 %)  EOS (7–10 days after last dose of medication)  Linezolid: 3/229(1.3 %);  Vancomycin: 6/210 (2.9 %) Microbiological outcome:  EOT (within 72 hours after discontinuation of medication):  Linezolid: 0/240 (0 %);  Vancomycin: 1/222 (0.5 %)  EOS (7–10 days after last dose of medication):  Linezolid: 1/229 (0.4 %);  Vancomycin: 1/210 (0.5 %) |
| Kohno 2007 [24] | NR | Open label | ITT population was used for safety (AE) analysis. Clinical response was reported in a subset of ME-MRSA patients who had cSSTI | a: NR  b: 7–21 days for pneumonia and cSSTI and 7–28 days for sepsis c: EOT and at the follow-up evaluation, 5–16 days post-treatment | NR |
| Lin 2008 [34] | NR | NR | ITT population was reported but not used for analyses relevant to this review. Randomly assigned patients who received at least one dose and conformed to a set of criteria were the analyzed population ("evaluable population") | a: Baseline (timing not specified) b: 7–21 days c: Up to 28 days post-treatment | Patients excluded from the "evaluable population":  EOT:  Linezolid: 0/33 (0 %)  Vancomycin: 3/29 (10.3 %)  EOS:  Linezolid: 0/33 (0 %)  Vancomycin: 5/29 (17.2 %), two of these five because of missing of indeterminate outcomes |
| Moran 2014 [[9](#_ENREF_9)] | Patients were randomly assigned (1:1) via an interactive voice-response system with block randomization. Randomization was stratified by geographic region and type of ABSSSI | Patients, study investigators, study staff and study sponsor were masked to treatment assignment  A double-dummy design was used with placebo unique to each active treatment | ITT analysis was conducted on all randomly assigned patients. Patients with missing data were considered treatment non-responders for the primary efficacy outcome | a: NR  b: 6 or 10 days c: 48- to 72-hour visit after first dose; EOT, which was on day 11 and post-therapy evaluation, which was 7–14 days after EOT and a late follow-up 18–25 days after last treatment | Tedizolid: 24/332 discontinued study drug; 290/332 were evaluable at PTE  Linezolid: 23/334 discontinued study drug; 280/334 were evaluable at PTE |
| Pertel 2009 [30] | Randomization was stratified by presence or absence of four complicating factors (diabetes mellitus, age ≥65 years, peripheral vascular disease, or an immunocompromising condition such as HIV infection) | Evaluator blinded. Additionally, a treatment-blinded physician reviewed concomitant medications and procedures received by each patient; if these were believed to have influenced the clinical outcome, the outcome was censored from the date of the procedure or medication administration | ITT population was not specified. 103 patients were randomly assigned, and 101 patients (all patients who received at least one dose of study medication) were included in the analyses. Data from patients who discontinued from the study prematurely were censored as of the last available evaluation | a: Baseline assessments were conducted within 3 days before the start of treatment  b: 7–14 days c: Evaluations were conducted three times per day, while patients were receiving study medication, 7–14 days after the last dose of study drug | All patients who received at least one dose of study medication were included in the analyses. "Data from patients who discontinued from the study prematurely were censored as of the last available evaluation" |
| Prokocimer 2013 [[10](#_ENREF_10)] | Randomization was stratified by presence or absence of fever at baseline, study center geographic region, and type of ABSSSI using block randomization via an interactive voice response system | Double blind | ITT analysis was conducted on all randomly assigned patients. There was also a safety analysis set of patients who were randomly assigned and received at least one dose of study drug and two further sets of patients who complied with the protocol until two different time points. Patients with missing data were considered treatment non-responders for the primary efficacy outcome | a: NR  b: 6 or 10 days c: 48- to 72-hour visit after first dose; EOT, which was on day 11, and post-therapy evaluation, which was 7–14 days after EOT | Tedizolid: 22/332 were lost to follow-up. In total, 43/332 (13.4%) were missing patient data  Linezolid: 21/335 were lost to follow-up. 28/335 (8.6%) were missing patient data |
| Sharpe 2005 [25] | NR | Open label | No ITT analysis was reported; however, all randomly assigned patients completed the prescribed therapy. No missing data were reported | a: NR  b: 7–21 days c: 10 days after EOT | Between-group comparisons were conducted using Fisher exact test |
| Stevens 2002 [26] | NR | Open label | ITT was conducted; however, the population included all randomly assigned patients who received one dose of study medication | a: NR  b: 7–14 days c: EOT and an indication-specific TOC (timing not reported) | "Missing" patients for clinical cure outcome (not reported for SSTI group for microbiological outcomes):  ITT SSTI:  Linezolid: 10/99 (10 %);  Vancomycin: 9/87 (10.3 %)  MRSA ITT SSTI:  Linezolid: 1/53 (1.9 %);  Vancomycin: 2/43 (4.7 %) Evaluable MRSA SSTI:  Linezolid: 0/34 (0 %);  Vancomycin: 2/30 (6.7 %) |
| Stryjewski 2008 [33] | Randomly assigned in a 1:1 ratio through an interactive voice-response system using a permuted block algorithm and stratified by geographic region and presence of diabetes mellitus | Double blinded. Site personnel involved in the evaluation of clinical response remained blinded to treatment assignment and serum vancomycin levels. A blinded core laboratory processed and analyzed all electrocardiograms | Reported to be ITT analysis, the analyzed population included all randomly assigned patients who received treatment (however, only 1867/1897 randomly assigned). For microbiological response, only ME patients were analyzed. No imputation of missing data | a: NR  b: 7–14 days c: EOT; within 72 hours after administration of last dose of study medication)  TOC; 7–14 days after administration of last dose of study medication | Lost to follow-up:  103/1897 (5.4 %) randomly  assigned.  565/1897 (29.7 %) patients were excluded from the analysis for other reasons |
| Talbot 2007 [31] | NR | Observer blinded (the investigators evaluating clinical response and safety were blinded to the treatment allocations) | No ITT analysis was performed. Analyses were undertaken on the mITT population; this population was used for safety analyses (AEs), clinical mITT, m-mITT, CE and ME populations | a: Baseline measures within 24 hours before drug administration b: 7–14 days (up to 21 days for severe infections) c: EOT, TOC 8–14 days after last dose of study drug, late follow-up 21–28 days after last dose of study drug | Loss to follow-up from mITT population:  Ceftaroline: 3/67 (4.5 %)  Vancomycin: 1/32 (3 %)  Other reasons for early discontinuation of study drug or withdrawal from study are reported by authors |
| Weigelt 2005 [27] | NR | Open label | ITT analysis was undertaken. Separate analyses were carried out on the mITT population, CE population, ME population and MRSA population | a: Baseline measures (timing not reported)  b: Minimum treatment period was 4 days and treatment duration was intended to be 7–14 days, but not >21 days c: TOC 7 days after EOT. All clinical end point measurements were completed at a maximum of 28 days from initial therapy | NR |
| Wilcox 2004 [28] | Study pharmacist/equivalent randomly assigned patients 1:1 to treatment arms upon enrollment after receiving randomization information and confirmation from the central randomization service (randomization list generated by Pharmacia)Randomization was not stratified | Open label. Some measurements used to assess efficacy (such as microbiological end points and changes in some vital signs) could be interpreted objectively and were to be evaluated by a blinded third party | ITT analysis was undertaken. However, patients with indeterminate and missing outcomes were excluded from the ITT for clinical outcome | a: Baseline screening b: 7–28 days  c: EOT within 72 hours of the last dose of study medication, STFU assessment scheduled 7–14 days after the EOT visit, and LTFU assessment, 15–21 days after the EOT visit, that was considered to be the TOC assessment | *Patients who discontinued during treatment and/or follow-up:  Linezolid: 43/215 (20 %)   Teicoplanin: 53/215 (25 %)* |
| Similarity assessment | 5/15 trials reported adequate randomization details, 2/15 trials (Aikawa 2013 [22] and Pertel 2009 [30]) did not report the randomization method and 8/15 trials did not provide any details | 4/15 trials reported double blinding. 7/15 trials were described as open label, although 2 of the 7 reported outcome assessor blinding in at least some outcomes. 2/15 trials reported outcome assessor blinding.  1/15 reported single blinding (NR if assessor, treatment administrator or patient)  1/14 did not report on the blinding | 3/15 trials assessed an ITT population, 6/15 assessed an mITT population who received at least a baseline amount of study medication, 1/15 assessed an "evaluable" population, 1/15 excluded patients who had missing/indeterminate outcomes, 2/15 assessed a per-protocol population and 2/15 lacked sufficient reporting on the population. Three studies reported using the ITT population for the safety analysis (AE data) and two studies reported using the mITT population. Three studies either lacked reporting or excluded patients with missing or indeterminate outcomes: (Evers 2013, Sharpe 2005 and Wilcox 2004) | When reported, baseline measures were taken within 3 days of the treatment. The treatment period ranged from 6 or 10 days (Prokocaimer 2013 and Moran 2014) to up to 28 days (Wilcox 2004; Florescu 2008), with the majority of studies reporting a treatment period of 7–14 days. Most studies reported follow-up either at the EOT (up to 72 hours after the last medication dose) or at the EOS, 7–37 days after the EOT | Loss to follow-up or patients missing/excluded for other reasons was generally <15% across all trials and treatment arms. Lin 2008 showed a skewed number of excluded patients between the linezolid (0 %) and vancomycin arms (10.3 % and 17.5 % at EOT and EOS). Larger percentages discontinuing treatment (20 % for linezolid and 25 % for teicoplanin) were reported by Wilcox 2004. Stryjewski 2007 excluded 29.7 % of patients from the analysis for reasons other than loss to follow-up |

*ABSSSI* acute bacterial skin and skin structure infections, *AE* adverse event, *APACHE* Acute Physiologic and Chronic Health Evaluation Scale, *CE* clinically evaluable, *cSSTI* complicated skin and soft tissue infection, *EOS* end of study, *EOT* end of treatment, *HIV* human immunodeficiency virus, *ITT* intention to treat, *LTFU* long-term follow-up, *ME* microbiologically evaluable, *mITT* modified intention to treat, *m-mITT* microbiological-modified intention to treat, *MRSA* methicillin-resistant *Staphylococcus aureus,* *PTE* post-treatment evaluation, *NR* not reported, *SSTI* skin and soft tissue infection, *STFU* short-term follow-up, *TOC* test of cure

**Table E.2** Confounding factors in relation to participant populations at baseline

*Data presented in italics within this table refer to a population including patients with infection types other than skin structure infections.*

| Study reference | Treatment arm | Eligible population age | Actual population age, mean (SD or range) | Gender, *n/N* (%) male | Ethnicity, *n/N* (%) | Mean weight (kg)/BMI (kg/m^2^) | Diagnostic workup |
| --- | --- | --- | --- | --- | --- | --- | --- |
| Aikawa 2013 [22] | Daptomycin | At least 20 years | Median (range) 69.0 (22–92) | 47/88 (53.4) | NR | Median  54.00 (28.3–117.8) | (1) Isolation of MRSA from specimens obtained within 3 days before starting treatment or the detection of gram-positive cocci and a strong suspicion of MRSA infection AND (2) presence of at least three of the following: drainage/exudate, erythema, fluctuance, localized warmth, pain/tenderness, swelling/induration, temperature >37.5 °C (oral) or 37 °C (armpit), out-of-normal-range white blood cell count, stab-cell >15%, pulse rate >90 beats/min, respiratory rate >20 breaths/min and positive C-reactive protein |
|  | Vancomycin |  | 70.0 (29–82) | 15/22 (68.2) |  | 52.25 (36.5–78.3) |  |
| Evers 2013 [29] | Daptomycin | NR | 69 | 10/20 [calculated] (50) | NR | NR | No tissue biopsies were obtained because all patients presented with only clinical cellulitis and SSTIs |
|  | Telavancin |  | 65 | 8/20 [calculated] (40) |  |  |  |
| Florescu 2008 [32] | Tigecycline | At least 18 years | *Median*  *51* | *76/117 (65)* | *White: 88/117 (75.2);*  *Black: 11/117 (9.4);*  *Hispanic: 16/117 (13.7);*  *Other: 2/117 (1.7)* | *80.1 ± 20.8* | Patients with a confirmed diagnosis of a serious infection (bacteremia, cIAI, cSSSI or pneumonia) requiring intravenous antibiotic therapy and were infected with vancomycin-resistant *Escherichia fecium* or *Escherichia fecalis* or MRSA, isolated alone or as part of a polymicrobial infection. cSSSIs included infections involving deep soft tissue, requiring significant surgical intervention or associated with a significant underlying disease state (diabetes mellitus, peripheral vascular disease, peripheral neuropathy or lower venous insufficiency) that complicates response to treatment. In addition to the infection, patients had to have at least two of the following signs and symptoms: drainage or discharge, fever, hypothermia, erythema, swelling, localized warmth, pain, white blood cell count >10 ×10^3^/ml, or immature bands >15 %. Infection in patients with a diabetic foot ulcer or decubitus ulcer had to be ≤1 week in duration. For this study, only data for patients with cSSSIs are included |
|  | Vancomycin |  | *51* | *23/39 (59)* | *White: 31/39 (79.5);*  *Black: 3/39 (7.7); Hispanic: 4/39 (10.3);*  *Other: 1/39 (2.6)* | *75.5 ± 20.0* |  |
| Itani 2010 [23] | Linezolid | At least 18 years | 49.7 (18–93) | 305/537 (57) | White: 361/537 (67);  Black: 91/537 (17) | 85.4 (36.0–295.5) | Symptoms of a cSSTI involving deep tissues and at least two of the following: purulent drainage, erythema, swelling or induration, tenderness or pain and local warmth. In addition, patients had to have at least one sign of systemic infection, such as fever, hypotension, increased white blood cell count (≥10,000 mm^3^), or >15% immature neutrophils regardless of the total peripheral white blood cell count. For patients with diabetic foot infections: evidence of a deep infection extending below the subcutaneous tissue of the foot, ankle or lower leg that required a surgical procedure. Patients with diabetic foot infections could be enrolled if there was an identifiable wound, such as an open lesion, ulcer or puncture wound, with evidence of purulence and either erythema in >50 % of the surface area of the foot, ankle and lower leg or medial arch streaking |
|  | Vancomycin |  | 49.4 (18–99) | 315/515 (61) | White: 352/515 (68);  Black: 89/515 (17) | 85.9 (42.0–244.1) |  |
| Kohno 2007 [24] | Linezolid | Over 20 years | *68.4 ± 16.4* | *70/100 (70)* | NR | *50.7 ± 12.8 (30.0*–*110.0)* | Body temperature >38 °C (oral) or >36 °C (axillary). Known or suspected MRSA infection, with signs and symptoms of active pneumonia, cSSTI or sepsis. Expected to survive study. cSSTI: signs and symptoms included erythema, swelling, fluctuation, drainage, tachycardia, hypotension or leukocytosis. Infections could include adnexal infection; diffuse infection; secondary infection of a burn, ulcer, abscess, external injury or post-operative wound. Carrier of MRSA, repetitive infection of MRSA, received an ineffective prior antibacterial agent, infection developed at least 48 hours after hospitalization |
|  | Vancomycin |  | *67.5 ± 16.3* | *36/51 (70.6)* |  | *53.0 ± 15.3 (29.8*–*107.0)* |  |
| Lin 2008 [34] | Linezolid | Aged 18–75 years | *56.3 ± 16.7* | *46/71 (64.8)* | NR | NR | Known or suspected infection due to gram-positive bacteria including MRSA, as determined by baseline culture results. Inclusion criteria for a known or suspected cSSTI that involved deeper soft tissue (such as deep and extensive cellulitis, major abscess or infected ulcer) included at least two of the following physical findings: drainage/discharge, erythema, fluctuance, heat/localized warmth, pain/tenderness to palpation or swelling/induration. In addition, all patients were required to have at least one of the following infection-related symptoms: fever, white blood cell count >10,000/mm^3^, or neutrophils >75 % |
|  | Vancomycin |  | *59.6 ± 13.3* | *42/71 (59.2)* |  |  |  |
| Moran 2014 [[9](#_ENREF_9)] | Tedizolid | At least 12 years | 46 (17–86) | 225 (68) | NR | Obesity:  101 (30 %) | Patients had ABSSSIs (cellulitis or erysipelas, major cutaneous abscess or wound infection) that had a minimum lesion area of 75 cm² and were suspected or documented to be associated with a gram-positive pathogen. Patients also had to have at least one systemic or regional sign of infection (lymphadenopathy, increased body temperature, white blood cell count ≥10 000/μl or <4000/μl or >10 % immature neutrophils) |
|  | Linezolid |  | 46 (15–89) | 214 (64) | NR | 118 (35 %) |  |
| Pertel 2009 [30] | Daptomycin | At least 18 years | Median (range)  57 (22–79) | 17/50 (34) | White: 40/50 (80):  Black: 7/50 (14);  Other: 3/50 (6.0) | Median BMI (range)  32 (20–82) | A primary diagnosis of cellulitis or erysipelas requiring hospitalization and intravenous antibiotic therapy was eligible for enrollment. The onset of symptoms and signs must have occurred within 3 days of the first dose of study medication, and a temperature >37.5 °C orally or >38.0 °C rectally had to be recorded within 48 hours before enrollment |
|  | Vancomycin |  | 55 (21–86) | 25/51 (49) | White: 36/51 (70.6);  Black: 12/51 (23.5);  Other: 3/51 (5.9) | 31 (18–55) |  |
| Prokocimer 2013 [[10](#_ENREF_10)] | Tedizolid | At least 18 years | 43.6 (SD 14.96) | 204/332 (61.4) | NR | NR | Patient had cellulitis/erysipelas, major cutaneous abscess or wound infection surrounded by erythema with a minimum total lesion surface area of 75 cm^2^ (measured head to toe, length × width), accompanied by at least one local and one regional (lymphadenopathy) or one systemic (oral temperature ≥38 °C, white blood cell count ≥10,000/µl or <4000/µl or >10 % of immature neutrophils) sign of infection, and a gram-positive pathogen was suspected or documented |
|  | Linezolid |  | 43.1 (15.06) | 198/335 (59.1) |  |  |  |
| Sharpe 2005 [25] | Linezolid | At least 18 years | 66 | 10/30 [calculated] (33) | NR | NR | At least one MRSA-infected skin lesion requiring surgical intervention. Biopsy and swab were two methods used for pathogen identification |
|  | Vancomycin |  | 76 | 15/30 [calculated] (50) |  |  |  |
| Stevens 2002 [26] | Linezolid | At least 13 years | *63.9 ± 16.1* | *143/240 (59.6)* | *White: 195/240 (81.3);*  *Black: 18/240 (7.5);*  *Other: 27/240 (11.3)* | *73.33 ±20.31* | Laboratory findings (e.g. Gram staining or culture results) consistent with *Staphylococcus aureus* infection and signs and symptoms consistent with SSTI. At least two of the following findings: drainage or discharge, erythema, fluctuance, heat or localized warmth, pain or tenderness to palpation, swelling or induration |
|  | Vancomycin |  | *59.8 ± 20.2* | *131/220 (59.5)* | *White: 168/220 (76.4);*  *Black: 30/220 (13.6);*  *Other: 22/220 (10.0)* | *73.10 ±-20.31* |  |
| Stryjewski 2008 [33] | Telavancin | At least 18 years | 48.8 *±* 16.6 | 517/928 (56) | White: 724/928 (78);  Black: 132/928 (14);  Asian: 45/928 (5) | BMI ≥30, *n/N* (%): 349/928 (38) | A diagnosis of cSSSI (caused by a suspected or confirmed gram-positive organism) that warranted ≥7 days of parenteral antibacterial therapy. cSSSI was defined by the presence of one of the following conditions: cellulitis, major abscess requiring surgical drainage, infected wound or ulcer or infected burn. Purulent drainage and/or collection or ≥3 of the following signs or symptoms also were required for participation: erythema, heat and/or localized warmth, fluctuance, swelling and/or induration, pain and/or tenderness to palpation, fever (temperature >38 °C), white blood cell count >10,000/mm^3^ or >15 % bands |
|  | Vancomycin |  | 48.7 *±* 16.6 | 559/939 (60) | White: 723/939 (77);  Black: 128/939 (14);  Asian: 53/939 (6) | 352/939 (37) |  |
| Talbot 2007 [31] | Ceftaroline | At least 18 years | 41.6 | 37/67 [calculated] (55.2) | Treatment groups were balanced | NR | SSSI requiring initial hospitalization and treatment with intravenous antimicrobials if the SSSI involved deeper soft tissue and/or required significant surgical intervention (e.g. surgical or traumatic wound infection, major abscess, infected ulcer or deep and extensive cellulitis) or had developed on a lower extremity in a subject with diabetes mellitus or well-documented peripheral vascular disease. Subjects were further required to have at least two local signs of cSSSI (purulent or seropurulent drainage/discharge, erythema, fluctuance, heat/localized warmth, pain/tenderness to palpation and swelling/induration) plus at least one systemic sign (oral temperature >38 °C, white blood cell count >10,000/mm^3^ and >10 % immature neutrophils) |
|  | Vancomycin |  | 44 | 19/32 [calculated] (59.4) |  |  |  |
| Weigelt 2005 [27] | Linezolid | NR | 51.7 *±* 17.6 | 375/592 (63.3) | White: 295/592 (49.8);  Black: 76/592 (12.8);  Asian: 138/592 (23.3);  Not disclosed: 83/592 (14.0) | NR | Suspected or proven MRSA infection that involved substantial areas of skin or deeper soft tissues, such as cellulitis, abscesses and infected ulcers or burns (<10 % of total body surface area). Required physical findings included: (1) erythema with or without induration, (2) fluctuance, (3) heat/localized warmth, (4) pain/tenderness and (5) drainage/discharge. In addition, all patients enrolled had at least one of the following symptoms: (1) fever, (2) hypothermia, (3) hypotension, (4) white blood cell count >10,000/mm^3^ or (5) >15 % immature neutrophils regardless of white blood cell count |
|  | Vancomycin |  | 51.8 *±* 18.0 | 363/588 (61.7) | White: 299/588 (50.9);  Black: 67/588 (11.4);  Asian: 136/588 (23.1);  Not disclosed: 86/588 (14.6) |  |  |
| Wilcox 2004 [28] | Linezolid | At least 13 years | *53 ± 20 (14*–*89)* | *117/215 (54)* | *White: 147/215 (68);*  *Black 4/215 (2); Asian/Pacific Islander: 2/215 (1) Mixed: 62/215 (29)* | *Mean; Median weight ± SD (lb)*  *167; 161 ± 47 (77*–*395)* | Confirmed or suspected gram-positive infection for which treatment with glycopeptide was clinically indicated, including pneumonia (hospital acquired or hospitalized after community acquired), severe SSTIs (infections that could involve the deeper levels [fascia, muscle] or extensive surface areas and required significant medical intervention and hospitalization), right-sided endocarditis or bacteremia |
|  | Teicoplanin |  | *55 ± 19 (14*–*97)* | *117/215 (54)* | *White: 145/215 (67);*  *Black: 5/215 (2); Asian/Pacific Islander: 2/215 (1);*  *Mixed: 63/215 (29)* | *162; 154 ± 41 (90–311)* |  |
| Similarity assessment | | Eligible ages were similar across studies, with minimum age of 18 or 20 years, two studies stated minimum age of 13, and one study, of 12 years | Actual ages were similar across studies, generally ~50 years, with a few studies up to 70 years | Percentage male was around 50–60 %, and values were similar across treatment arms | When reported, similar proportions of whites (50–80 %), blacks (2–-25 %) and Asian/other races were reported by studies | In the two Japanese trials by Aikawa 2013 and Kohno 2007, body weights were ~55 kg, compared with body weights reported by other trials, which were ~75 kg and mean BMI of ~30. This is likely to reflect the ethnic difference | Diagnostic workup was generally well reported. Differences between studies mostly reflect differences in the population groups assessed by studies, e.g. studies that required a suspected or proven MRSA infection, e.g. Weigelt 2005, and those that used only clinical signs of SSSI (e.g. Evers 2013) as diagnostic tools |

*ABSSSI* acute bacterial skin and skin-structure infection, *BMI* body mass index, *cIAI* complicated intra-abdominal infection, *cSSSI* complicated skin and skin structure infection, *cSSTI* complicated skin and soft tissue infection, *MRSA* methicillin-resistant *Staphylococcus aureus,* *NR* not reported, *SD* standard deviation, *SSTI* skin and soft tissue infection

**Table E.3** Confounding factors in relation to participant populations at baseline (continuation table)

*Data presented in italics within this table refer to a population including patients with infection types other than skin structure infections.*

| Study reference | Treatment arm | Details of infection, *nN* (%) | Details of pathogen identified at baseline | Severity of condition |
| --- | --- | --- | --- | --- |
| Aikawa 2013 [22] | Daptomycin | Deep skin infection (including cellulitis): 6/88 (6.8, calculated); Wound or burn: 38/88 (43.2, calculated);  Erosion or ulcer: 9/88 (10.2, calculated); Other infection: 2/88 (22.7, calculated) | Isolation of MRSA from specimens obtained within 3 days before starting treatment or the detection of gram-positive cocci:  55/88 (62.5 %, calculated) | NR  Inclusion criteria require presence of at least three of the following: drainage/exudate, erythema, fluctuance, localized warmth, pain/tenderness, swelling/induration, temperature >37.5^o^C (oral) or 37^o^C (armpit), out-of-normal-range white blood cell count, stab-cell >15%, pulse rate >90 beats/min, respiratory rate >20 breaths/min and positive C-reactive protein |
|  | Vancomycin | Deep skin infection (including cellulitis): 0/22 (0); Wound or burn: 13/22 (59.1); Erosion or ulcer: 5/22 (22.7); Other infection: 1/22 (4.5) | 19/22 (86.4 %, calculated) |  |
| Evers 2013 [29] | Daptomycin | NR | NR | Average white blood cell count (cells/cu/L):  7.5 |
|  | Telavancin |  |  | 8.9 |
| Florescu 2008 [32] | Tigecycline | *NR 81/117 (69.2) were cSSSI* | Confirmed MRSA as a baseline isolate (i.e. repeat positive culture required if patients had received a recent antibiotic prior to study entry) | *APACHE II score, patients classified as either >15 or ≤15  Mean ± SD: 7.9 ± 5.0* |
|  | Vancomycin | *27/39 (69.2) were cSSSI* |  | *7.9 ± 6.3* |
| Itani 2010 [23] | Linezolid | Abscess: 243/537 (45); Surgical wound infection: 111/537 (21); Diabetic ulcer: 61/537 (11); Other infected skin ulcer: 44/537 (8); Other: 34/537 (6); Trauma wound infection: 26/537 (5); Decubitus ulcer: 11/537 (2); Infected burn: 7/537 (1) | Culture-proven MRSA in the MRSA ITT population | Wilson severity score for the MRSA ITT population. The Wilson severity score is a validated scoring system in which baseline variables predict outcome: 31.8 ± 1.4 (*n* = 322) |
|  | Vancomycin | Abscess: 238/515 (46); Surgical wound infection: 123/515 (24); Diabetic ulcer: 45/515 (9); Other infected skin ulcer: 41/515 (8); Other: 30/515 (6); Trauma wound infection: 23/515 (5); Decubitus ulcer: 8/515 (2); Infected burn: 7/515 (1) |  | 31.2 ± 1.3 (*n* = 318) |
| Kohno 2007 [24] | Linezolid | *NR 31/100 (31) were cSSSI* | *MRSA detected: 71/100 (71 %)* | Defined as cSSTI |
|  | Vancomycin | *17/51 (33.3) were cSSSI* | *34/51 (66.7 %)* |  |
| Lin 2008 [34] | Linezolid | NR | *Staphylococcus aureus 25/31 (80.6 %, calculated)  Enterococcus spp. 4/31 (12.9 %, calculated)  Streptococcus spp. 2/31 (6.5 %, calculated)* | Defined as cSSTI involving deeper soft tissue and present with at least two physical and one infection related symptom |
|  | Vancomycin |  | *S. aureus 16/24 (66.7 %, calculated) Enterococcus spp. 5/24 (20.8 %, calculated) Streptococcus spp. 3/24 (12.5 %, calculated)* |  |
| Moran 2014 [[9](#_ENREF_9)] | Tedizolid | Cellulitis/erysipelas: 166 (50)  Major cutaneous abscess: 68 (20)  Wound: 98 (30) | MRSA: 53 (27 %)  MSSA: 105 (53 %) | NR; patients with ABSSSI  Inclusion criteria require presence of cellulitis/erysipelas, major cutaneous abscess, or wound infection surrounded by erythema with a minimum total lesion surface area of 75 cm^2^ (measured head to toe, length × width), accompanied by at least one local and one regional (lymphadenopathy) or one systemic (oral temperature ≥38 ^o^C, white blood cell count ≥10 000/µl or <4000 µl or >10% immature neutrophils) sign of infection |
|  | Linezolid | Cellulitis/erysipelas: 168 (50)  Major cutaneous abscess: 68 (20)  Wound: 98 (29) | MRSA: 56 (28 %)  MSSA: 111 (55 %) |  |
| Pertel 2009 [30] | Daptomycin | Leg: 40/50 (80.0); Arm: 5/50 (10.0);  Other: 5/50 (10.0) | NR | Temperature, median (range): 37.4 ^o^C (35.3–39.8 ^o^C); Symptoms and signs composite score, median (range): 6 (1–13);  Patient-reported pain score, median (range): 45.5 (1.0–100.0); Patient-reported tightness/swelling score, median (range): 63.0 (1.0–100.0) |
|  | Vancomycin | Leg: 38/51 (74.5);  Arm: 4/51 (7.8);  Other: 9/51 (17.6). |  | Temperature, median (range): 37.2 ^o^C (35.6 –39.2 ^o^C); Symptoms and signs composite score, median (range): 6 (1–12); Patient-reported pain score, median (range): 73.0 (0.0–100.0); Patient-reported tightness/ swelling score, median (range): 70.0 (2.0–100.0) |
| Prokocimer 2013 [[10](#_ENREF_10)] | Tedizolid | Cellulitis/erysipelas: 135/332 (40.7); Major cutaneous abscess: 100/332 (30.1); Wound: 97/332 (29.2) | *S. aureus*; MRSA; MSSA | NR; patients with ABSSSI  Inclusion criteria require presence of cellulitis/erysipelas, major cutaneous abscess or wound infection surrounded by erythema with a minimum total lesion surface area of 75 cm^2^ (measured head to toe, length × width), accompanied by at least one local and one regional (lymphadenopathy) or one systemic (oral temperature ≥38 ^o^C, white blood cell count ≥10 000/µl or <4000 µl or >10% immature neutrophils) sign of infection |
|  | Linezolid | Cellulitis/erysipelas: 139/335 (41.5); Major cutaneous abscess: 98/335 (29.3); Wound: 98/335 (29.3) |  |  |
| Sharpe 2005 [25] | Linezolid | Foot or calf cSSSI:  21/30, calculated (70) | Infection with MRSA was confirmed by biopsy in 80 % of infections and by swab in the other 20 %. Other isolated pathogens included enterococci (*n* = 6) and gram-negative organisms (*n* = 6). One patient was infected with MRSA, enterococci and gram-negative bacteria | NR |
|  | Vancomycin | 18/30, calculated (60) |  |  |
| Stevens 2002 [26] | Linezolid | Infected surgical incision or wound: 49/92 (53.3); Skin ulcer, abscess or other lesion: 31/92 (33.7); Cellulitis: 12/92 (13.0); Area of skin lesion >28 cm^2^: 44/86 (51.2) | *Culture-proven infection with a staphylococcal pathogen: 157/240 (65.4 %, calculated); S. aureus isolated: 124/240 (51.7 %, calculated); Confirmed MRSA infection: 117/240 (48.8 %, calculated).* | Inclusion criteria state hospitalized patients were eligible, suggesting complicated infection |
|  | Vancomycin | Infected surgical incision or wound: 45/83 (54.2); Skin ulcer, abscess or other lesion: 28/83 (33.7);  Cellulitis: 10/83 (12.0);  Area of skin lesion >28 cm^2^: 37/76 (48.7) | *Culture-proven infection with a staphylococcal pathogen: 144/220 (65.5 %, calculated); S. aureus isolated: 118/220 (53.6 %, calculated); Confirmed MRSA infection: 107/220 (48.6 % calculated)* |  |
| Stryjewski 2008 [33] | Telavancin | Abscess: 388/928 (42); Cellulitis: 335/928 (36); Wound infection: 144/928 (16); Infected ulcer: 45/928 (5); Infected burn: 16/928 (2) | Identified from primary site of infection  Gram-positive organism: 639/673 (95 %);  *S. aureus*: 553/673 (82 %)   MRSA: 348/673 (52 %)  *Enterococcus faecalis*: 32/673 (5 %);  *Enterococcus* spp.: 9/673 (1 %);  *Streptococcus pyogenes*: 28/673 (4 %);  *Streptococcus agalactiae*: 22/673 (3 %);  *Streptococcus anginosus*: 14/673 (2 %);   *Streptococcus dysgalactiae*: 7/673 (1 %);  Gram-negative organism: 114/673 (17 %) | Fever (>38 °C), *n* (%): 126/928 (14) White blood cell count >10 ×10^9^; *n* (%): 224/928 (35) |
|  | Vancomycin | Abscess: 402/939 (43); Cellulitis: 356/939 (38); Wound infection: 124/939 (13); Infected ulcer: 48/939 (5); Infected burn: 9/939 (<1) | Identified from primary site of infection:  Gram-positive organism: 666/698 (95 %);  *S. aureus*: 590/698 (85 %);  MRSA: 369/698 (53 %);  *E. faecalis*: 43/698 (6 %);  *Enterococcus* spp.: 4/698 (<1 %);  *S. pyogenes*: 33/698 (5 %);  *S. agalactiae*: 22/698 (3 %);  *S. anginosus*: 8/698 (1 %);  *S. dysgalactiae*: 10/698 (1 %);  Gram-negative organism: 110/698 (16 %) | Fever (>38 °C), *n* (%): 145/939 (15) White blood cell count >10 ×10^9^, *n* (%): 347/939 (37) |
| Talbot 2007 [31] | Ceftaroline | Major abscess: 30/67 (44.8); Deep extensive cellulitis: 23/67 (34.3); Infected wound: 7/67 (10.4); Lower-extremity cSSSI: 4/67 (6.0) ; Infected ulcer: 1/67 (1.5); Infected bite: 0/67 (0.0); Other: 2/67 (3.0) | MRSA: 6/67 (9.0 %) | Defined as cSSTI  Inclusion criteria state patients with SSSI requiring initial hospitalization and treatment with intravenous antimicrobials were eligible if the SSSI involved deeper soft tissue and/or required significant surgical intervention |
|  | Vancomycin | Major abscess: 16/32 (50.0); Deep extensive cellulitis: 12/32 (37.5); Infected wound: 1/32 (3.1); Lower-extremity cSSSI: 0/32 (0.0); Infected ulcer: 0/32 (0.0); Infected bite: 2/32 (6.3); Other: 1/32 (3.1) | 6/32 (18.8 %) |  |
| Weigelt 2005 [27] | Linezolid | Cellulitis: 282/592 (47.6); Major skin abscess: 158/592 (26.7); Infected ulcer: 40/592 (6.8); Infected burn: 7/592 (1.2); Infected surgical incision: 63/592 (10.6);  Infected traumatic wound: 14/592 (2.4);  Other: 28/592 (4.7). | MRSA: 142/592 (41.3 %); MSSA: 106/592 (30.8 %) | Defined as cSSTI  MPM severity score (mean). A validated MPM II scoring system: 3.5 |
|  | Vancomycin | Cellulitis: 266/588 (45.2); Major skin abscess: 146/588 (24.8); Infected ulcer: 39/588 (6.6); Infected burn: 12/588 (2.0); Infected surgical incision: 65/588 (11.1); Infected traumatic wound: 22/588 (3.7); Other: 38/588 (6.5) | MRSA: 146/588 (44.1 %); MSSA: 95/588 (28.7 %) | 3.6 |
| Wilcox 2004 [28] | Linezolid | *83 % of the skin/soft tissue lesions in SSTI patients were characterized as deep/extensive lesions*  *Linezolid: 123/215 were SSSI;*  *Teicoplanin: 117/215 were SSSI* | *261/430 (61 %) ITT patients had at least one pathogen isolated at baseline, of which 84.7% had a gram-positive pathogen. S. aureus was isolated from infection sites in 58 % and 50 % of linezolid and teicoplanin patients, respectively, who had at least one baseline pathogen* | *The distribution of illness severity was very similar between the two treatment groups in each of the infection types. Baseline screening included severity of illness scoring using standard systemic inflammatory response syndrome criteria* |
|  | Teicoplanin |  |  |  |
| Similarity assessment | | There is variability between trials in proportions of patients with different infection types, each varying from ~10 % up to 50 %. Trials that included patients with non–skin structure infections did not report the infection details | Trials varied in the proportion of patients with confirmed MRSA; this variation depended on the focus of the trial on MRSA or on all causes of infection | Although not reported in all trials, severity of infection was generally similar between treatment groups  The majority of trials refer to complicated or acute skin and skin structure or soft tissue infections |

*APACHE* Acute Physiologic Assessment and Chronic Health Evaluation, *cSSSI* complicated skin and skin structure infection, *cSSTI* complicated skin and soft tissue infection, *ITT* intention to treat, *MPM* mortality probability model, *MRSA* methicillin-resistant *Staphylococcus aureus*; *MSSA* methicillin-sensitive *Staphylococcus aureus*, *NR* not reported, *SSSI* skin and skin structure infection, *SSTI* skin and soft tissue infection

**Table E.4** Confounding factors in relation to participant populations at baseline (continuation table)

*Data presented in italics within this table refer to a population including patients with infection types other than skin structure infections.*

| Study reference | Treatment arm | Details of prior therapy | Concomitant therapy | Co-existing disease | Other relevant baseline characteristics |
| --- | --- | --- | --- | --- | --- |
| Aikawa 2013 [22] | Daptomycin | 10/88 (11.4 %) daptomycin patients and 2/22 (9.1 %) vancomycin patients had an anti-MRSA antibiotic within 28 days before the start of the study drug administration. Patients had no previous systemic antimicrobial therapy for >24 hours during the previous 3 days (unless unresponsive to at least 72 hours’ antibiotic therapy or detection of a resistant pathogen) | No concomitant therapies were reported | NR. Patients with osteomyelitis, infectious arthritis, known or suspected pneumonia or HIV were excluded | Creatinine clearance rate ≥80 mL/min: 41/88 (46.6 %) |
|  | Vancomycin |  |  |  | Creatinine clearance rate ≥80: 10/22 (45.5 %) |
| Evers 2013 [29] | Daptomycin | NR | NR | 10/20, calculated (50 %) had diabetes but did not have significant vascular insufficiency as observed by palpable bilateral dorsalis pedis pulses | NR |
|  | Telavancin |  |  | 8/20, calculated (40 %) had diabetes |  |
| Florescu 2008 [32] | Tigecycline | NR | Patients were excluded if in receipt of >24 hours of potentially effective concomitant antibacterial therapy for MRSA after baseline culture was obtained, but before the first dose of the study drug | Patients were excluded if they had any concomitant condition | *Creatinine clearance, mean ± SD (ml/min): 112.1 + 55.7* |
|  | Vancomycin |  |  |  | *116.8 ± 50.2* |
| Itani 2010 [23] | Linezolid | Patients previously treated with an antibiotic with activity against the MRSA isolate (including linezolid, vancomycin and teicoplanin) for >24 hours within or spanning any part of the 72 hours before enrollment were excluded | Patients who took concomitant medication with activity against the patient’s isolate had their result set to failure | Type 1 or 2 diabetes: 34 % | Treatment groups were well balanced with respect to demographic and baseline characteristics |
|  | Vancomycin |  |  | Type 1 or 2 diabetes: 32 % |  |
| Kohno 2007 [24] | Linezolid | The CE, mITT or ME-MRSA populations did not receive antibiotic therapy before the start of study medication that continued during the study (prior antibiotic use that stopped at study start was acceptable). Patients with cSSTI or sepsis may have had an ineffective prior antibacterial agent | Patients in the CE, mITT or ME-MRSA populations did not receive potentially effective concomitant antibiotic(s) against MRSA for an adverse event or intercurrent illness during the study period | NR | NR |
|  | Vancomycin |  |  |  |  |
| Lin 2008 [34] | Linezolid | No patients in the cSSTI group had antibiotic treatment within 48 hours of baseline unless the treatment failed or the pathogen showed drug resistance | Patients in the cSSTI subgroup did not receive concomitant antibiotics (unless otherwise allowed) | *Tumor: 19/71 (26.8 % calculated) CNS disease: 12/71 (16.9 %, calculated)) Diabetes mellitus: 2/71 (2.8 %, calculated)) Respiratory disease: 11/71 (15.5 %, calculated)) Multiple wound: 2/71(2.8 %, calculated))* | NR |
|  | Vancomycin |  |  | *Tumor: 17/71 (23.9 %, calculated) CNS disease: 18/71 (23.4 %, calculated) Diabetes mellitus: 3/71 (4.2 %, calculated) Respiratory disease: 16/71 (22.5 %,calculated) Multiple wound: 1/71 (1.4 %, calculated)* |  |
| Moran 2014 [[9](#_ENREF_9)] | Tedizolid | Patients receiving systemic or topical antibiotics with gram-positive activity <96 hours before the first dose of study drug were excluded | NR | Diabetes: 10 %  Obesity: 30 %  Renal impairment: 4 %  Hepatitis C: 20 %  Concurrent secondary ABSSSI lesion: 14 % | Region of enrollment:  North America 47 %  Latin America 4 %  Europe 34 %  South Africa 14 %  Australia and New Zealand 1 % |
|  | Linezolid |  |  | Diabetes: 12 %  Obesity: 35 %  Renal impairment: 4 %  Hepatitis C: 25 %  Concurrent secondary ABSSSI lesion: 14 % | Region of enrollment:  North America 47 %  Latin America 4 %  Europe 33 %  South Africa 14 %  Australia and New Zealand 2 % |
| Pertel 2009 [30] | Daptomycin | 32.0 % of daptomycin- and 35.3% of vancomycin-treated patients had a previous episode of cellulitis or erysipelas | A similar proportion of patients received concomitant medications or underwent procedures that could have influenced outcomes. At least one dose of a systemic antibiotic other than the assigned study medication was received by 44.0 % of daptomycin- and 51.0 % of vancomycin-treated patients. One patient (2.0%) in the daptomycin group and three patients (5.9 %) in the vancomycin group underwent incision and drainage procedures within the past 5 years. Anti-inflammatory drugs were administered to 28.0 % (14/50) and 29.4 % (15/51) of daptomycin- and vancomycin-treated patients, respectively. One daptomycin-treated patient received at least 1 day of topical steroid treatment for the infection and one vancomycin-treated patient received at least 4 days of systemic steroid therapy | Diabetes: 15/50 (30 %); Peripheral vascular disease: 14/50 (28 %) | Immunocompromised condition: 0 (0 %) |
|  | Vancomycin |  |  | Diabetes: 11/51 (21.6 %) Peripheral vascular disease: 8/51 (15.7 %) | Immunocompromised condition: 1/51 (2.0 %) |
| Prokocimer 2013 [[10](#_ENREF_10)] | Tedizolid | Patients receiving systemic or topical antibiotics with gram-positive activity <96 hours before the first dose of study drug were excluded | NR | Hepatitis C: 101/329 (30.7 %) | Region of enrollment:  North America 270/332 (81.3 %);  Latin America 9/332 (2.7 %);  Europe 53/332 (16 %). "Current or recent intravenous drug use" 117/332 (35.2 %) |
|  | Linezolid |  |  | Hepatitis C: 116/327 (35.5 %) | Region of enrollment:  North America 268/335 (80 %);  Latin America 12/335 (3.6 %);  Europe 55/335 (16.4 %)  "Current or recent intravenous drug use": 132/335 (39.4 %) |
| Sharpe 2005 [25] | Linezolid | NR | Concomitant topical and systemic antibiotic treatments that were ineffective for MRSA were allowed | NR | NR |
|  | Vancomycin |  |  |  |  |
| Stevens 2002 [26] | Linezolid | Patients were excluded if they had 24 hours of potentially effective antibiotic within 48 hours of study entry (unless that therapy had failed or the isolated pathogen was drug-resistant) | Concomitant administration of aztreonam or gentamicin was allowed, concomitant systemic corticosteroid therapy was not. >50% of enrolled patients received a concomitant monoamine oxidase inhibitor–interacting drug. Concomitant aminoglycoside use in patients with suspected gram-negative pathogens was rare | NR | NR |
|  | Vancomycin |  |  |  |  |
| Stryjewski 2008 [33] | Telavancin | Patients were excluded if they had received prior antibiotic therapy for >24 hours within 7 days before enrollment (unless the pathogen was resistant to therapy or the patient had experienced clinical failure after 3 days of systemic antibiotic therapy) | Aztreonam: 297/928 (32 %); Metronidazole: 213/928 (23 %) | Diabetes: 231/928 (24.9 %) | Impaired renal function (estimated creatinine clearance <50 ml/min): 114/928 (12 %) |
|  | Vancomycin |  | Aztreonam: 313/939 (33 %); Metronidazole: 205/939 (22 %) | Diabetes: 233/939 (24.8 %) | 111/939 (12 %) |
| Talbot 2007 [31] | Ceftaroline | Patients with more than a single prior dose of a non-study antimicrobial within the 96 hours before randomization were excluded, unless there was clear evidence of failure | In the vancomycin/cloxacillin group, if the presence of a gram-negative pathogen was suspected at baseline, concomitant administration of aztreonam (1 g every 8 hours) was allowed | Some patients had diabetes mellitus or a well-documented peripheral vascular disease | NR |
|  | Vancomycin |  |  |  |  |
| Weigelt 2005 [27] | Linezolid | NR | Concomitant use of aztreonam or other antibiotics for gram-negative organisms was permitted. Patients who were receiving another investigational medication concurrently were excluded | NR | Geographic region:  Asia Pacific: 126 (21.3 %);   South America: 54 (9.1 %);   North America: 366 (61.8 %);  Europe and New Zealand: 46 (7.8 %) |
|  | Vancomycin |  |  |  | Geographic region:  Asia Pacific: 125 (21.3 %);  South America: 64 (10.9 %);  North America: 351 (59.7 %);  Europe and New Zealand: 48 (8.2 %) |
| Wilcox 2004 [28] | Linezolid | Patients were excluded if they had previously been enrolled in a linezolid trial | Patients were excluded if they had concurrent use of another investigational medication. 127/430 (47 linezolid/80 teicoplanin) received at least one concomitant antibiotic during and/or after treatment with the study medication. 88/430 (30 linezolid/58 teicoplanin) of these patients received one or more antibiotic approved for concomitant use by the protocol. 36/430 (14 linezolid/22 teicoplanin) received potentially effective antibiotics for gram-positive pathogens, other than the study medication, of sufficient duration and timing to confound the assessment of clinical success that were considered potentially effective against the relevant gram-positive pathogens. Differences in use of concomitant antibiotics between the teicoplanin and linezolid treatment groups were not significantly different | NR | NR |
|  | Teicoplanin |  |  |  |  |
| Comparability assessment | | Previous potentially effective antibiotic treatment was generally reported as an exclusion criterion | Itani 2010 stated that concomitant therapy necessitated results setting to zero. Most other trials allowed some degree of concomitant therapy or use of some permitted concomitant antibiotics. Systemic steroid therapy was not permitted in the Stevens 2002 trial, and one patient in the Pertel 2009 trial took systemic steroids | Diabetes was a commonly reported co-existing disease, varying in proportions from 4–50 %. Most diseases were in similar proportions between treatment arms | Region of enrollment and creatinine clearance rates were reported as other baseline characteristics. Values were generally similar between treatment arms and across studies |

*ABSSSI* acute bacterial skin and skin-structure infection, *CE* clinically evaluable, *CNS* central nervous system, *cSSTI* complicated skin and soft tissue infection, *HIV* human immunodeficiency virus, *ME* microbiologically evaluable, *mITT* modified intention to treat, *MRSA* methicillin-resistant *Staphylococcus aureus*, *NR* not reported, *SD* standard deviation

**Table E.5** Confounding factors in relation to circumstances

| Study reference | Location(s) | Setting | Date of trial |
| --- | --- | --- | --- |
| Aikawa 2013 [22] | Japan (61 centers). | 61 Japanese medical institutions | Between 2008 and 2010 |
| Evers 2013 [29] | NR | NR | NR |
| Florescu 2008 [32] | 50 sites in 14 countries | NR | Between November 2003 and August 2005 |
| Itani 2010 [23] | 102 centers in the United States (*n* = 640; 61 %), Eastern and Western Europe (*n* = 172; 16 %), Latin America (*n* = 122; 12 %), and South Africa, Malaysia, and Singapore (*n* = 118; 11 %) | NR | Between October 2004 and July 2007 |
| Kohno 2007 [24] | 84 sites in Japan | Hospital (named within article) | Between October 2001 and January 2004 |
| Lin 2008 [34] | 7 hospitals in China | Hospital | Between April 2001 and March 2005 |
| Moran 2014 [[9](#_ENREF_9)] | 58 centers in nine countries (Argentina, Australia, Germany, New Zealand, Poland, Russia, South Africa, Spain, and United States) | NR | Between September 2011 and January 2013 |
| Pertel 2009 [30] | 15 sites in United States, South Africa and Serbia | NR | NR |
| Prokocimer 2013 [[10](#_ENREF_10)] | North America, Latin America and Europe (81 study centers) | NR | Between August 2010 and September 2011 |
| Sharpe 2005 [25] | Single center | NR | NR |
| Stevens 2002 [26] | 104 sites in North America, Europe, Latin America and Asia | NR | Between July 1998 and July 1999 |
| Stryjewski 2008 [33] | 129 centers in 21 countries, ~69 % of patients in United States, 31 % outside the United States | NR | Between January 2005 and June 2006 |
| Talbot 2007 [31] | 15 clinical sites in United States, South America, South Africa and Russia | Clinical sites | NR |
| Weigelt 2005 [27] | Multinational, multicenter | NR | Between October 2003 and March 2003 |
| Wilcox 2004 [28] | 50 investigator sites in 13 countries in Europe and Latin America | Investigator sites | Between November 1999 and December 2000 |
| Comparability assessment | Two studies were carried out in Japan, one in China and 10 were multinational, when reported, mostly from Westernized countries, though South America, Latin America and Asia were also included. Sharpe 2005 [25] was single-center (unreported location) and Evers 2013 [29] did not report the location | The majority of studies did not report the setting, those that did reported medical sites, hospitals and clinical or investigator sites | When reported, most trials were conducted post 2000, the latest ending in January 2013. Two earlier trials began in November 1999 and July 1998 |

*NR* not reported

**E.2 Similarity assessment of common treatment arms**

**Table E.6** Similarity assessment of linezolid treatment arms of eligible studies

| Study reference | Dose and schedule | Duration | Method of administration |
| --- | --- | --- | --- |
| Itani 2010 [23] | 600 mg every 12 hours | 7–14 days | Oral or intravenous, with the option to switch from the intravenous to the oral formulation at any time at the investigator’s discretion |
| Kohno 2007 [24] | 600 mg | 7–21 days for cSSTI | Intravenous or oral |
| Lin 2008 [34] | 600 mg every 12 hours | 7–21 days | Intravenous |
| Prokocimer 2013 [[10](#_ENREF_10)] | 600 mg every 12 hours | 10 days | Oral tablet |
| Sharpe 2005 [25] | 600 mg every 12 hours | 7–21 days | Oral |
| Stevens 2002 [26] | 600 mg twice daily | At least 7 days, recommended for 7–14 days | Intravenous. When they had shown clinical improvement, linezolid-treated patients could be switched to oral at the discretion of the investigator |
| Weigelt 2005 [27] | 600 mg | The minimum treatment period was 4 days and treatment duration was intended to be 7–14 days, but not >21 days | Intravenous or oral |
| Wilcox 2004 [28] | 600 mg every 12 hours | 7–28 days | Intravenous or intravenous with a switch to oral |
| Similarity assessment | Dose was 600 mg every 12 hours in all cases in which dosing schedule was reported | Treatment duration was generally 7–21 days, though shorter in four studies and longer in one study, up to 28 days | Linezolid was administered either intravenously or orally, in some trials both orally and intravenously, and in other trials, orally before intravenously in the same patient |

*cSSTI* complicated skin and soft tissue infection

**Table E.7** Similarity assessment of vancomycin treatment arms of eligible studies

| Study reference | Dose and schedule | Duration | Method of administration |
| --- | --- | --- | --- |
| Aikawa 2013 [22] | 1 g over at least 60 minutes; twice daily | 7–14 days | Intravenous |
| Florescu 2008 [32] | Intravenous dose of 1 g approximately every 12 hours. Doses could be adjusted for patients with compromised renal function | 7–28 days, depending on the site and severity of infection and was based on the investigator’s judgment | Intravenous |
| Itani 2010 [23] | 15 mg/kg every 12 hours with dose adjustment as necessary, based on trough levels and creatinine clearance | 7–14 days | Intravenous |
| Kohno 2007 [24] | 1 g every 12 hours | 7–21 days for cSSTI | Intravenous |
| Lin 2008 [34] | 1 g if aged ≤60 years or 0.75 g if aged >60 years, every 12 hours | 7–21 days | Intravenous |
| Pertel 2009 [30] | Administered "according to standard of care" | 7–14 days | Intravenous |
| Sharpe 2005 [25] | 1 g every 12 hours | 7-21 days | Intravenous |
| Stevens 2002 [26] | 1 g twice daily | At least 7 days, recommended for 7–14 days | Intravenous |
| Stryjewski 2008 [33] | 1 g every 12 hours. Individualized vancomycin dosing was permitted (including adjustment using serum vancomycin level monitoring), as dictated by standard practice at the individual participating site | 7–14 days | Intravenous |
| Talbot 2007 [31] | 1 g every 12 hours | 7–14 days, up to 21 days for subjects with a severe infection that required extended intravenous antibiotic therapy, but only with the approval of the medical monitor for the study. Mean length of therapy was 8.0 days (range 2.0–20.5 days) | Intravenous |
| Weigelt 2005 [27] | 1 g every 12 hours | Minimum treatment period was 4 days and treatment duration was intended to be 7–14 days, but not >21 days | Intravenous |
| Similarity assessment | Dosing and scheduling were similar across trials. In most trials, the dose was 1 g every 12 hours. One study, Itani 2010, dosed according to a patient's weight. At least three trials report that dosing could be adjusted | Treatment duration was generally 7–14 days, though up to 21–28 days in four studies | In all trials, vancomycin was administered intravenously |

*cSSTI* complicated skin and soft tissue infection

**Table E.8** Similarity assessment of daptomycin treatment arms of eligible studies

| Study reference | Dose and schedule | Duration | Method of administration |
| --- | --- | --- | --- |
| Aikawa 2013 [22] | 4 mg/kg over 30 minutes; once daily | 7–14 days | Intravenous |
| Evers 2013 [29] | 4 mg/kg/day (average 5.6 mg/kg/day) | Average of 10–14 days | NR |
| Pertel 2009 [30] | 4 mg/kg once daily | 7–14 days | Intravenous |
| Similarity assessment | Dosage was the same across all studies | Treatment duration was similar; up to 14 days in all trials | When reported in two of three trials, daptomycin was administered intravenously. |

*NR* not reported

**Table E.9** Similarity assessment of telavancin treatment arms of eligible studies

| Study reference | Dose and schedule | Duration | Method of administration |
| --- | --- | --- | --- |
| Evers 2013 [29] | 10 mg/kg/day (average 8.2 mg/kg/day) | 10–14 days | NR |
| Stryjewski 2008 [33] | 10 mg/kg every 24 hours | 7–14 days | Intravenous |
| Similarity assessment | Dosage was the same across all studies | Treatment duration was similar; up to 14 days in all trials | Not possible to assess. |

*NR* not reported

**E.3 Definition of response as reported in the included trials**

**Table E.10** Definition of responder/clinical success in the trials reporting data

| Study reference | Definition of responder/clinical success |
| --- | --- |
| Aikawa 2013 [[21](#_ENREF_21)] | A successful clinical response had to meet all the following criteria: an EAC-confirmed clinical response of ‘‘cured’’ (resolution of clinically significant signs and symptoms) or ‘‘improved’’ (partial resolution of clinically significant signs and symptoms) at both EOT and TOC; the patient did not receive a non-study antibacterial agent that could potentially have been effective against the causative pathogen; ≥4 days of study drug administration to the patient |
| Evers 2013 [[28](#_ENREF_28)] | Response demonstrated by resolution of the cellulitis and reduction of the pain and erythema |
| Florescu 2008 [[31](#_ENREF_31)] | Response was determined by the principal investigator. Responders: defined as resolution of signs and symptoms or improvement such that no further antibacterial therapy was required |
| Itani 2010 [[22](#_ENREF_22)] | Responders were patients who were cured or showed improvement. Non-responders were patients with treatment failure. Investigator outcome was defined as the clinical assessment recorded in the case report form |
| Kohno 2007 [[23](#_ENREF_23)] | Responders were patients who were cured or improved. Cured was defined as resolution of the clinical signs and symptoms of infection when compared with baseline; improved was defined as improvement in two or more, but not all, clinical signs and symptoms of infection when compared with baseline |
| Lin 2008 [[33](#_ENREF_33)] | Resolution in at least three of four areas identified at baseline as abnormal: (1) signs, (2) symptoms, (3) hematology and chemistry and (4) microbiology |
| Moran 2014 [[9](#_ENREF_9)] | Patients were classed as responders if they had a ≥20 % reduction in area (length × width of erythema, edema and induration) of the primary lesion from baseline, did not receive any systemic concomitant antibiotics with gram-positive activity and did not die of any cause within 72 hours of the first dose |
| Pertel 2009 [[29](#_ENREF_29)] | A patient cured or improved. Definition of responder was unclear. Assume investigator assessment of symptoms and signs (based on a composite score of three symptoms: tenderness, chills and warmth) |
| Prokocimer 2013 [[10](#_ENREF_10)] | Investigator’s assessment of clinical success at the post-treatment evaluation in the ITT analysis and responders were patients who had cessation of primary ABSSSI lesion spread compared with baseline, did not receive prohibited concomitant antibiotics and did not die of any cause |
| Sharpe 2005 [[24](#_ENREF_24)] | Clinical cure was defined as temperature normalization, presence of granulation or wound healing, resolution of pain and decreased or resolved erythema, edema, induration and color. Ulceration could persist, but lesions must appear uninfected to be defined as clinical cure. Clinical improvement was defined as moderate resolution of two or more clinical symptoms |
| Stevens 2002 [[25](#_ENREF_25)] | “Cure” was defined as resolution of baseline clinical signs and symptoms of infection after ≥5 days and ≥10 doses of treatment |
| Stryjewski 2008 [[32](#_ENREF_32)] | Response was defined as resolution of clinically significant signs and symptoms associated with cSSSI present at study admission or improvement to the extent that the infectious process had been controlled and no further antimicrobial therapy was necessary |
| Talbot 2007 [[30](#_ENREF_30)] | Response was determined by a blinded investigator  Resolution of all signs and symptoms of the cSSSI or improvement of the infection such that no further antimicrobial therapy was necessary |
| Weigelt 2005 [[26](#_ENREF_26)] | Complete resolution of all pre-therapy clinical signs and symptoms of infection (e.g. body temperature and white blood cell count) was achieved. Patients were considered improved if two or more (but not all) pre-therapy clinical signs and symptoms of cSSTI were resolved. Response was judged by the investigator |
| Wilcox 2004 [[27](#_ENREF_27)] | Response assessed by the investigator to be resolution of clinical signs and symptoms of gram-positive infection, when compared with baseline (e.g. body temperature and white blood cell count). Improvement or lack of progression of infection-related radiographic abnormalities OR improvement in two or more, but not all, clinical signs and symptoms of gram-positive infection, when compared with baseline |
| Similarity assessment | All trials stated that patients with a resolution or improvement in key clinical symptoms or a cessation of spread were classed as responders. While there were differences in the specific definitions between trials, they were deemed similar for purposes of comparison |

*ABSSSI* acute bacterial skin and skin structure infection, *cSSTI* complicated skin and soft tissue infection, *EAC* efficacy adjudication committee, *EOT* end of treatment, *ITT* intention to treat, *TOC* test of cure

**E.4 Analysis populations defined in the included trials**

**Table E.11** Population definition in the trials reporting data

*Populations in italics indicate those that featured in the analyses.*

| Study reference | Population | Definitions of analysis populations |
| --- | --- | --- |
| Aikawa 2013 [22] | *mITT with confirmed MRSA culture* | *mITT-MRSA included all patients who received at least one dose of study drug and in whom MRSA infection was confirmed on screening cultures* |
| Evers 2013 [29] | *ITT population* | *ITT population included all randomly assigned patients who received at least one dose of study medication* |
| Florescu 2008 [32] | *Microbiological mITT* | *Patients in the mITT population who had clinical evidence of disease by meeting minimal disease criteria (i.e. they satisfied all a priori requirements for cSSSIs) and had confirmed VRE or MRSA as a baseline isolate (i.e. repeat positive culture required if patients had received a recent antibiotic prior to study entry)* |
|  | ME population | ME population includes those who met inclusion and exclusion criteria, received no more than one dose of potentially effective concomitant therapy for MRSA or VRE infection after the first dose of the study drug, received no more than 24 hours of concomitant therapy between obtaining the baseline culture and starting the study drug, had a clinical response of cure or failure at the TOC assessment and a pre-therapy culture containing MRSA or VRE that was susceptible to both of the relevant study drugs and microbiological or clinical information to allow classification of a microbiological response at the TOC assessment |
| Itani 2010 [23]  Kohno 2007 [24] | *mITT* | *The mITT population included all ITT patients who had a positive culture at screening* |
|  | PP population | The PP population, defined separately at each visit, included mITT patients who took ≥80 % of the required study medication for the first 7 days without missing more than two consecutive doses, adhered to adjunctive therapeutic procedure guidelines and had all their visits within the visit window |
| Lin 2008 [34] | ME population | *ME population included all patients in the CE population (population who met enrollment criteria, did not receive antibiotic therapy before the start of study medication that continued during the study, received study medication for a minimum of 2 days and four doses if a clinical failure or 5 days and 10 doses if a clinical cure, were at least 80% compliant with study medication, did not receive potentially effective concomitant antibiotic[s] against MRSA for an adverse event or intercurrent illness during the study period and had required follow-up assessment) with culture-confirmed Staphylococcus aureus at baseline and baseline pathogen was not resistant to study medications* |
| Moran 2014 [[9](#_ENREF_9)] | *Evaluable population* | *The evaluable population consisted of patients in the mITT group who fulfilled inclusion criteria, received at least 80% of prescribed study medication, returned for required follow-up visits, had at least 5 days of study medication for effective treatment or at least 72 hours of study medication for failed treatment and did not receive concomitant antibiotics* |
| Itani 2010 [23] | *ITT population* | *ITT: all randomly assigned patients* |
| Pertel 2009 [30] | *ITT population* | *All patients who received study drug* |
| Prokocimer 2013 [[10](#_ENREF_10)] | *ITT population* | *ITT: all randomly assigned patients* |
|  | CE population | Patients in the CE-EOT/CE-PTE analysis set completed the relevant assessments without major protocol violations or receiving treatments that might confound outcomes |
| Sharpe 2005 [25] | *ITT population* | *ITT population included all randomly assigned patients who received at least one dose of study medication* |
| Stevens 2002 [26] | *ITT population* | *ITT population included all randomly assigned patients who received at least one dose of study medication* |
|  | *ITT population with confirmed MRSA culture* | *MRSA-ITT population was the subset of MITT patients with culture and susceptibility testing results that confirmed infection with an MRSA isolate at baseline* |
| Stryjewski 2008 [33] | *ITT population* | *ITT population included all treated patients* |
|  | CE population | CE population was defined as patients in the all-treated population who complied with all exclusion and inclusion criteria and had a clinical response of either cure or clinical failure at TOC |
| Talbot 2007 [31] | CE population | CE population included all subjects in the cmITT population who had no confounding factors that interfered with the assessment of outcome. In addition, subjects in the CE population must have received 80–120 % of the intended study drug doses, received at least 48 hours (for failure evaluation) or 96 hours (for success evaluation) of therapy, and had the outcome assessment performed within 7–20 days after the end of therapy or were determined to be a clinical failure at an earlier time point |
|  | *mITT population with confirmed cSSSI* | *cmITT population comprised all subjects in the mITT population with a confirmed cSSSI* |
| Weigelt 2005 [27] | ITT population | ITT population included all treated patients |
|  | *mITT population* | *mITT population, which included all ITT patients who had a culture-confirmed gram-positive pathogen at baseline* |
|  | CE population | CE population included all patients who received >4 days of therapy and returned for the TOC visit |
|  | ME population | ME population included all CE patients who had one or more gram-positive pathogen at baseline that was not resistant to the study drug |
| Wilcox 2004 [28] | *ITT population* | *ITT population included all randomly assigned patients who received at least one dose of study medication* |

*CE* clinically evaluable, *cSSSI* complicated skin and skin structure infection, *cmITT* all subjects in the mITT population with a confirmed cSSSI, *EOT* end of treatment, *ITT* intention to treat, *ME* microbiologically evaluable, *MRSA* methicillin-resistant *Staphylococcus aureus,* *mITT* modified intention to treat, *MITT* microbiological intention to treat, *PP* per protocol; *PTE* post-treatment evaluation, *TOC* test of cure, *VRE* vancomycin-resistant *Enterococcus*

# APPENDIX F Results of the random-effects analyses

**Table F.1** Random-effects results of NMA comparing tedizolid with each of the seven comparator drugs (odds ratios and 95 % credible intervals)

| Outcome | Comparator drug | | | | | | |
| --- | --- | --- | --- | --- | --- | --- | --- |
|  | Ceftaroline | Daptomycin | Linezolid | Teicoplanin | Telavancin | Tigecycline | Vancomycin |
| Clinical response at the EOT | | | | | | | |
| All trials | 0.9 [0.0, 48.2] | NA | 1.0 [0.5, 2.1] | 2.3 [0.4, 14.2] | NA | NA | 2.0 [0.8, 7.1] |
| ITT/mITT only | NA | NA | 1.0 [0.4, 2.3] | 2.3 [0.4, 15.7] | NA | NA | 1.5 [0.4, 6.6] |
| Clinical response at PTE/TOC | | | | | | | |
| All trials | 1.1 [0.2, 9.2] | 1.7 [0.4, 9.0] | 1.0 [0.4, 2.4] | NA | 1.7 [0.4, 9.0] | 3.9 [0.5, 39.0] | 1.9 [0.7, 6.5] |
| ITT/mITT only | 1.2 [0.1, 13.6] | 1.7 [0.3, 12.6] | 1.0 [0.3, 2.9] | NA | 1.7 [0.3, 13.4] | 4.1 [0.5, 54.7] | 2.0 [0.6, 9.2] |
| MRSA only | NA | 2.9 [0.0, 230.3] | 1.0 [0.1, 14.3] | NA | 1.4 [0.0, 93.3] | 4.5 [0.1, 350.1] | 2.2 [0.1, 52.4] |
| Post hoc sensitivity analysis | | | | | | | |
| MRSA only | 2.4 [0.1, 257.9] | 2.9 [0.0, 265.5] | 1.0 [0.1, 16.1] | NA | 1.4 [0.0, 114.5] | 4.5 [0.1, 409.1] | 2.2 [0.1, 60.0] |
| Discontinuation due to AE | | | | | | | |
| All trials | 0.3 [0.0, 16.6] | 0.8 [0.0, 83.3] | 0.5 [0.1, 2.7] | NA | 0.3 [0.0, 4.9] | NA | 0.4 [0.0, 4.5] |

*AE* adverse event, *EOT* end of treatment, *ITT* intention to treat, *mITT* modified intention to treat, *MRSA* methicillin-resistant *Staphylococcus aureus*, *NA* not available, *PTE* post-treatment evaluation, *TOC* test of cure

1. When reported, data will be collected for both microbiological and clinical definitions of “cure.” [↑](#footnote-ref-1)
